# Spatiotemporal dynamics of the oropharyngeal microbiome in a cohort of Ivorian school children

**DOI:** 10.1038/s41598-024-81829-6

**Published:** 2024-12-28

**Authors:** K. Diallo, K. F. Missa, K. J. Tuo, L. S. Tiemele, A. F. Ouattara, K. D. T. Gboko, B. G. Gragnon, K. B. Bla, J. M. Ngoi, R. J. Wilkinson, G. A. Awandare, B. Bonfoh

**Affiliations:** 1 Centre Suisse de Recherches Scientifiques en Côte d’Ivoire (CSRS), Abidjan, Côte d’Ivoire; 2 West African Centre for Cell Biology of Infectious Pathogens (WACCBIP), Accra, Ghana; 3 Laboratoire de Biologie et Santé, UFR Biosciences, Université Félix Houphouët Boigny de Cocody (UFHB), Abidjan, Côte d’Ivoire; 4 Laboratoire de Microbiologie, Biotechnologies et Bio-informatique, Institut National Polytechnique Félix Houphouët-Boigny, Yamoussoukro, Côte d’Ivoire; 5 Laboratoire de Cytologie et Biologie Animale, Université Nangui Abrogoua, Abidjan, Côte d’Ivoire; 6 Laboratoire National d’Appui au Développement Agricole (LANADA), Laboratoire Régional de Korhogo, Korhogo, Côte d’Ivoire; 7 The Francis Crick Institute, London, NW1 1AT UK; 8 Department of Infectious Diseases, Imperial College London, London, W12 0NN UK; 9 Centre for Infectious Diseases Research in Africa, Institute of Infectious Disease and Molecular Medicine and Department of Medicine, University of Cape Town, Observatory, Cape Town, 7925 Republic of South Africa

**Keywords:** Oropharyngeal microbiome, *Neisseria* carriage, Meningococcal, Microbial diversity, Côte d’Ivoire, DNA sequencing, Meningitis, Microbial genetics

## Abstract

The respiratory tract harbours microorganisms of the normal host microbiota which are also capable of causing invasive disease. Among these, *Neisseria* meningitidis a commensal bacterium of the oropharynx can cause meningitis, a disease with epidemic potential. The oral microbiome plays a crucial role in maintaining respiratory health. An imbalance in its composition is associated with increased risk of invasive disease. The main objective of this study was to evaluate changes in the spatio-temporal dynamics of the oropharyngeal microbiota considering meningococcal carriage in a cohort of 8–12-year-old school children within (Korhogo) and outside (Abidjan) of the meningitis belt of Côte d’Ivoire. A significant geographic difference in the oropharyngeal microbiome was identified between the two study sites in terms of bacterial abundance and diversity (*p* < 0.001), with greater diversity in children in Abidjan than in Korhogo. Meningococcal carriage was low in the cohort with eight *Neisseria* carriers identified in Korhogo (3.64%) including one *Neisseria meningit*idis (0.45%). No *Neisseria* were detected in Abidjan indicating geographical differences in carriage (*p* = 0.006). Negative correlations were also found between *Neisseria* abundance and humidity. Meningococcal carriage was very low during the study; however, *Neisseria* carriage differed between the two study areas, with a higher frequency in children in Korhogo. Analysis of the oropharyngeal microbiome showed significant differences between children followed in Abidjan and Korhogo with higher microbial diversity in Abidjan, which is generally associated with better health status. Significant correlations between *Neisseria* or other pathogens carriage and climatic variables (Temperature, Relative humidity, and Wind speed) were also demonstrated, indicating an important role of climate in the carriage of these bacteria; an important element to note in the current context of climate change.

**Supplementary Information:**

The online version contains supplementary material available at 10.1038/s41598-024-81829-6.

## Introduction

Meningitis is a severe infectious disease characterised by inflammation of the protective layer of the brain and cerebrospinal spaces. It can be caused by different types of pathogens, but bacterial meningitis is the most dangerous with its capacity to cause local outbreaks or epidemics. Three main pathogens implicated in bacterial meningitis are: *Neisseria meningitidis* (*N. meningitidis*), *Streptococcus pneumoniae* (*S. pneumoniae*) and *Haemophilus influenzae* (*H. influenzae)*^1^. Despite being able to cause invasive disease, these 3 bacteria are also part of the normal human oropharyngeal microbiota and are transmitted between humans through contact with infected droplets via coughing, kissing, or other exchange of nasopharyngeal fluids^2^. The progression from being an asymptomatic carrier to a patient with invasive meningitis is not fully understood and is likely to be multi-factorial, involving bacteria and human genetic factors as well as environmental ones^3^.

Meningococcal meningitis remains an important public health concern, especially in the African meningitis belt where 20 843 cases were reported in 2018, with a case fatality rate of 7.2%^4^. The region is characterised by recurrent meningococcal disease epidemics every 7–10 years. However, the introduction of serogroup A conjugate vaccine in multiple countries of the region in 2010, resulted in lower sized outbreaks of different aetiology^5–7^. One of the most striking features of meningococcal meningitis in this region is its seasonality, with epidemics starting during the dry/cold/windy season (Harmattan) between October/ December and ending with the onset of rains around April/May^8,9^ with a peak in number of cases in February/March^10^.

Although several hypotheses have been proposed, the underlying reasons for this seasonality are not yet understood. The pharyngeal microbiome has been shown to play a crucial role in maintaining respiratory health, and imbalances in its composition associate with increased risk of diseases like pneumonia^11^ or cystic fibrosis associated lung disease^12^. However, little data is available for the role that it may play in meningitis.

Carriage is known to be a prerequisite for development of bacterial meningitis and therefore it is important to monitor carriage of the most common bacteria involved in meningitis to inform public health responses. Carriage also represents more accurately bacterial population diversity as only a few strains are known to be involved in invasive cases^13,14^.

Most carriage studies to date have used culture-based methods to characterise the meningococcus^15,16^. Among these, some have examined other bacteria such as non-pathogenic *Neisseria* species and have found an inverse relationship between the presence of *N. meningitidis* and the other commensals^15^. Human challenge experiments with *N. lactamica* have also shown that carriage of the latter can prevent colonisation by the potential pathogen^17,18^. This raises questions on the dynamics of the oropharyngeal bacterial carriage which require unbiased methods like metagenomics to answer.

Two metagenomic approaches are available: hypervariable region of the 16 S ribosomal RNA gene sequencing based on the diversity of the variable loops of the ribosomal genes 16 S, known to be present in all bacteria, and the shotgun whole genome metagenomic approach which relies on parallel sequencing of all genetic information present in a sample and bioinformatic identification of different bacterial species. Both represent an improvement in term of sensitivity for bacterial detection when compared to culture methods with the most sensitive method being whole genome metagenomic sequencing^19,20^.

Many African countries have part of their territory within the meningitis belt and other parts outside of that region. However, it is important to note that the majority of carriage studies have been conducted within areas of the meningitis belt and little attention was paid to bacterial carriage in regions less prone to epidemics outside of the belt. Côte d’Ivoire is one such example; with the northern territories characterised by a highly variable seasonal climate, prone to regular meningitis epidemics, whereas the south of the country has a more constant and humid climate with lower risk of epidemics^21^. These distinct regions provide an ideal opportunity to study the effect of seasonal variation on the pharyngeal microbiome and assess how such differences could influence susceptibility to meningococcal infection, and its progression to invasive meningococcal disease.

This study characterized seasonal changes in the oropharyngeal microbiome, including *N. meningitidis* carriage, and associated changes in local mucosal immunity in a cohort of Ivorian school children (8–12 years old) in the northern and southern parts of the country.

## Methodology and analysis

### Study sites and participants

**Fig. 1:**
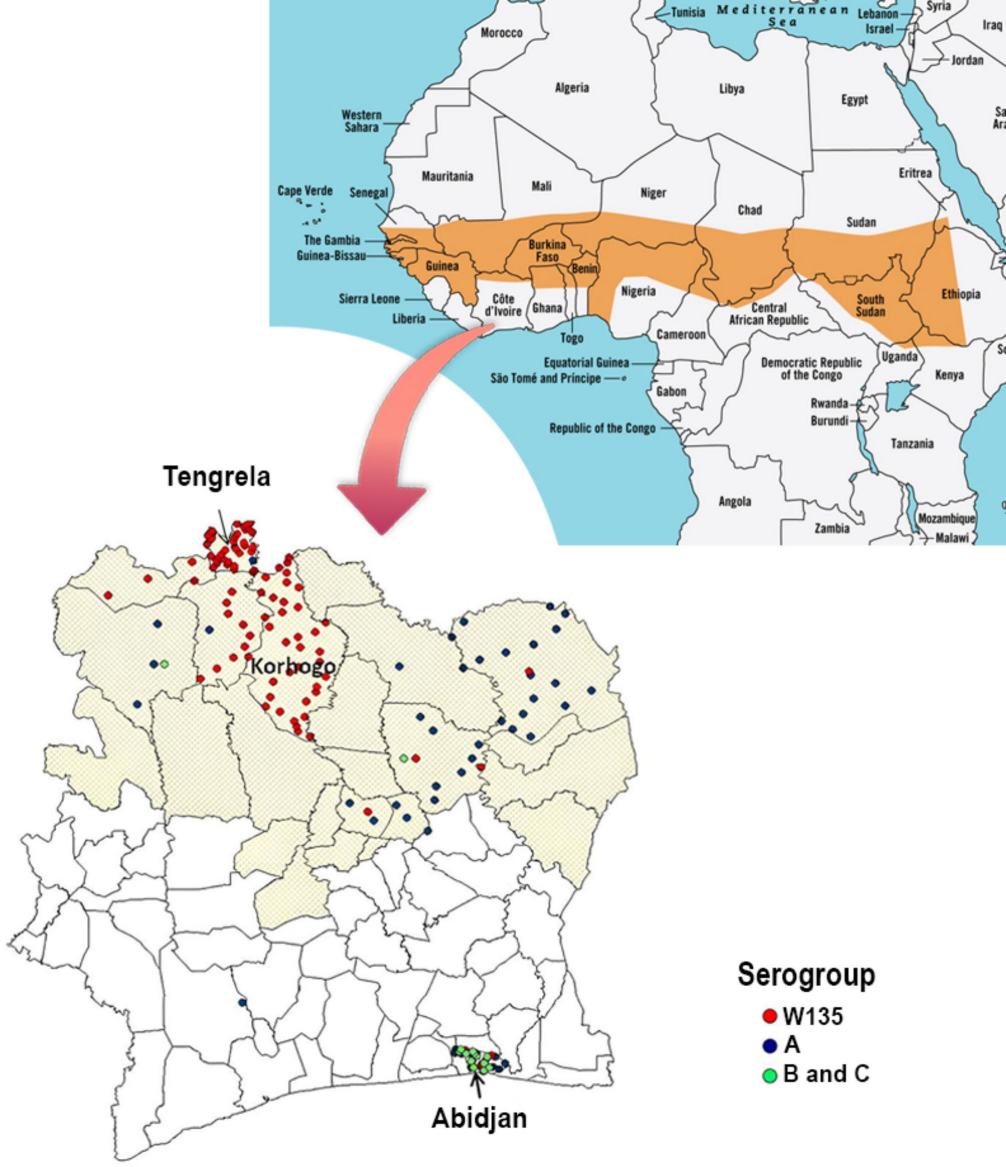
Map of Côte d’Ivoire in the context of the African meningitis belt. The orange shape in the map above presents the location of the African meningitis belt; the zoom on Côte d’Ivoire shows with better precision the regions of the country within that African meningitis belt (in light yellow). The city in which the two sites have been selected for this study are highlighted. The spatial distribution of *Neisseria meningitidis* serogroups A, W135 and B/C received at the National Reference Centre for meningitis between 2007 and 2012 is also shown. This figure was modified from one published in 2018 by Soumahoro et al.^21^ This is an open-access article distributed under the terms of the Creative Commons Attribution License (http://creativecommons.org/licenses/by/4.0/), which permits unrestricted use, distribution, and reproduction in any medium, provided the original work is properly cited.

Two local public primary schools were included in the study: one in Korhogo within the meningitis belt and the second one in the southern part of the country in Abidjan (Fig. 1). Schools were selected on the basis that they were public primary schools situated in urban areas within less than 5 km of the laboratories where samples would be processed. Additionally, the school director’s willingness to provide an up-to-date list of students from the previous school year was required. In Korhogo 10 schools were identified sharing the same characteristics and the closest one to the LANADA laboratory was chosen. In Abidjan, only 1 school met al.l these criteria. A list of school attendants aged 8–12 years old was generated from the registration files and used to randomly select and invite children to participate to the study. This age range was identified as the most susceptible to meningococcal carriage in previous studies conducted in the African meningitis belt^3^. Children were only enrolled in the study after informed consent was obtained from their parents and assent obtained from children 10 years and older in accordance with the ethical approval from the national ethical committee (Ethical approval number: IRB000111917). Based on the literature and cost of sequencing an optimum number of 30 participants per site was required; however, a maximum of 40 participants per site (total of 80) was sought-after, taking into account the possibility of consent retraction and loss to follow up estimated at 20% due to the longitudinal nature of the project.

### Questionnaires

A questionnaire was designed using Open Data Kit (ODK)^22^ to collect demographic data and information about factors that may influence the pharyngeal microbiome and risk of meningococcal carriage. It was administered once, at enrolment, and responses were given by the parents or legal representative of each participant. (Supplemental Table 1).

### Samples collection and processing

Two oropharyngeal swabs and one saliva sample were collected from each participant at each monthly Survey (S1-S6) between November 2020 and April 2021.

The oropharyngeal samples were collected with a FLOQSwabs flexible swab with a nylon tip (Copan) using standard methods described elsewhere^23^. The first swab was plated directly in the field on Thayer-Martin agar plates and incubated upon arrival at the laboratory between 16 and 48 h at 37 °C in 5% CO_2_.

The second swab was stored in 1 ml of RNA protect in the field; vortexed upon arrival at the laboratory and aliquoted into 3 cryovials of 250 µl swab wash each for downstream analysis.

A maximum of 1 ml saliva sample was collected by the passive drooling method (Salimetrics). Children were asked to rinse their mouth, and a minimum of 10 min later were asked to accumulate saliva for 1 min before dropping the saliva in the collection tubes. This process was repeated until 1 ml was obtained, and the number of minutes. The saliva was aliquoted and stored at − 80 °C, for immunological analyses.

All experiments were performed in accordance with relevant guidelines and regulations.

### 16 S sequencing for bacterial microbiome

DNA was extracted from an aliquot of the second swab, using the DNeasy PowerSoil kit following manufacturers procedures (Qiagen). The DNA concentration was assessed using the qubit DNA concentration kits (HS and BS). The DNA concentrator kit (Zymo) was used to increase the concentration of the samples by reducing the elution buffer.

### 16 S PCR

The V3-V4 hypervariable region of the 16 S rRNA gene was targeted for sequencing. Forward and reverse primers targeting this region were generated with an Illumina adapter overhang sequence appended to the primer pair 341 F/785R 16 S^24^ for compatibility with Illumina index and sequencing adapters. Amplifications were done in 25 µl reactions with 12.5 µl Q5 Hot Start High-Fidelity 2X Master Mix (NEB), 5 µl of 1µM forward and reverse 16 S primer and 2.5 µl of template. The reactions were carried out on ABI Veriti thermocyclers (Applied Biosytems) under the following conditions: 95 °C for 3 min, 25 cycles of; 95 °C for 30 s, 55 °C for 30 s, 72 °C for 30 s, followed by 72 °C for 5 min and a final hold at 4 °C. The amplified products were then verified on 1.5% agarose gel with a product of ~ 550 bp expected.

### 16 S library preparation and sequencing

All samples irrespective, including controls of whether an amplification band was visible or not were sent for library preparation and sequencing. The amplified products were further purified using Agencourt AMPure XP beads (BeckmanCoulter) for library preparation. Libraries were then prepared by ligating Illumina dual indices and Illumina sequencing adapters to the purified amplicons using the NexteraXT index kit. Attachment of the indices was performed using 5 µl of the 16 S amplicon DNA, 5 µl of Illumina Nextera XT Index Primer 1 (N7xx), 5 µl of Nextera XT Index Primer 2 (S5xx), 25 µl of Q5 Hot Start High-Fidelity 2X Master Mix (NEB), and 10 µl of PCR-grade water (Ambion). The reactions were carried out on ABI Veriti thermocyclers (Applied Biosytems) under the following conditions 95 °C for 3 min, followed by 8 cycles of 95 °C for 30 s, 55 °C for 30 s, and 72 °C for 30 s, a final extension at 72 °C for 5 min and a final hold at 4 °C. The libraries were then purified using Agencourt AMPure XP beads (BeckmanCoulter) and thereafter size distribution and library quality control performed using the Agilent 2100 Bioanalyzer (Agilent) to confirm the expected size distribution and quality. The libraries were finally quantified using the Qubit dsDNA HS kit on the Qubit 4.0 fluorometer (Life Technologies) normalized and pooled at equimolar concentration based on the Qubit results. A total of 10pM of the pooled library was then spiked with 8% Phix (v3) for sequencing. Sequencing was done on the Illumina MiSeq system using 2 × 300 bp PE sequencing with the MiSeq Reagent Kit v3 (600 cycle).

### Characterisation of *Neisseria*

All Gram negative dipococci and oxidase positive bacteria were subject to classical biochemical analysis to identify *Neisseria* species based on their ability to hydrolyse particular molecules. Bacteria were classified as *N. meningitidis* if they were positive to the Gama Glutamyl Aminopeptidase (GGA) test; *N. lactamica* if positive in the O-nitrophenyl ß-galactoside (ONPG) test; *Moraxella* if they were positive in the tributyrin test and *Neisseria spp* if they were Gram negative, oxidase positive but negative in GGA, ONPG and tributyrin tests^23,25,26^. DNA was extracted from all cultured Gram negative, oxidase positive diplococci bacteria using the Promega Wizard DNA extraction kit and stored at -80 °C for whole genome sequencing.

### Molecular confirmation of *Neisseria* carriage

The remaining DNA extracted from the second swab (used for 16 S sequencing) was used for molecular characterisation of *N. meningitidis* using a real time multiplex PCR assay detecting *N. meningitidis*, *S. pneumoniae* and *H. influenzae.* The assay used primers from the *ctrA* gene (F753/R846) as target to detect the meningococcus, *Hel* and *SP 2020* for *H. influenzae* and *S. pneumoniae*^27,28^.

The amplifications were done in 25 µl reactions with 12.5 µl Invitrogen Platinum SuperMix (Thermofisher), 1.5 µl of MgCl_2_, 1 µl of 2.5µM forward and reverse primers from each pair, 1 µl of 2 µM of each probe and 2 µl of template. The reactions were carried out on a Biorad thermocycler (Biorad) under the following conditions: 50 °C for 2 min, 95 °C for 10 min, 45 cycles of; 95 °C for 15 s followed by 60 °C for 1 min and a final hold at 4 °C. CT values ≤35 were considered positives.

### Measure of mucosal immunity

Total salivary secretory immunoglobulin A (SIgA, µg/mL) was measured using the salivary secretory IgA indirect enzyme immunoassay kit (Salimetrics) following the manufacturer’s guidelines. Concentrations were calculated from the optical densities using a Four Parameter Logistic Fit (4PL) and the specified dilution factor of 5 using a Salimetrics “secretory immunoglobulin A” template on a software freely available on the website http://www.myassays.com^29^.

### Climatic data

Three climatic data information: temperature at 2 m (in degree celsius, °C), wind speed at 10 m (meter per second, m/s) and relative humidity at 2 m (%) were downloaded from the Prediction of Worldwide Energy Resource (POWER) database of the American National Aeronautics and Space Administration (NASA) using the Data Access Viewer tool^30,31^. Geographical coordinates of the two schools involved in the study and dates of samples collection were used as input information.

### 16 S RNA bioinformatic and statistical analysis

#### Data processing and analysis

Raw sequences were processed using the DADA2 pipeline (Divisive Amplicon Denoising Algorithm)^32^. Quality filtering and denoising were performed with the filterAndTrim() function, applying the following parameters: truncLen = c(270,270) for forward and reverse reads, trimLeft = 20, maxN = 0, maxEE = c(5,10), and truncQ = 2. Error rates were modeled using the learnErrors() function, applied to the filtered forward (filtFs) and reverse (filtRs) reads. The resulting error models were used for denoising the data.Subsequently, sequences were dereplicated using derepFastq(), denoised with dada(), and merged using mergePairs(). Chimeric sequences were removed with the removeBimeraDenovo() function to ensure only true variants were retained. The resulting amplicon sequence variants (ASV) were classified by taxonomy and mapped to a reference set of operational taxonomic units (OTU) at 99% sequence similarity using the Silva database (Silva_nr_v138)^33^ with the assign-Taxonomy() function. ASVs were processed using the DADA2 pipeline, which includes a taxonomic classification step. The ASVs were aggregated to higher taxonomic levels (phylum, genus, and species) for the analysis of microbial relative abundances and diversity metrics. The data were pruned to ensure the robustness of the dataset. First, we removed taxa with total abundances ≤ 10 across all samples to reduce noise. Next, we excluded samples with zero total abundance and retained those with a total abundance ≥ 1000 to focus on complete and relevant data. Finally, we eliminated taxa that had zero abundance in the remaining samples to finalize the dataset. Blank samples (negative controls) were included to monitor potential contamination during the sample preparation process. Positive control samples containing *Escherichia coli* DNA were used to validate the sequencing pipeline. Contaminants were detected in the blank samples, and the decontam package^34^ was applied to both blank and experimental samples. This tool identifies potential contaminants by comparing the prevalence of sequence features in true samples versus blanks, based on presence/absence across samples. The data was then combined into a phyloseq object.

### Statistical analysis

The Shannon index, β-diversity and relative abundances (at phylum and genus level) were performed using the phyloseq package^35^. Prior to calculating the Shannon index for alpha-diversity, the data were rarefied to a depth of 30,000 reads per sample to ensure comparability across samples. For abundance and beta-diversity analyses, however, non-rarefied data were used to avoid information loss and maintain the accuracy of relative abundance estimates. Wilcoxon rank sum test, or Kruskal-Wallis H test with pairwise comparison using Dunnett test were used to compare immunological data and microbial diversity in two groups or more than two groups, respectively. Non-metric multidimensional scaling (NMDS) was conducted using the metaMDS function from the R package *vegan*^34^, with Bray–Curtis dissimilarities calculated after Hellinger transformation^36^ to compare similarities among samples. The association between the microbiome and factors such as geographical location, gender, and visits was tested using permutational multivariate analysis of variance (PERMANOVA) via the *vegan* package^34^. To assess the variability within groups, we conducted a Permutational Analysis of Multivariate Dispersions (PERMDISP) using the vegan package^34^. Bray-Curtis distances were used to quantify the dissimilarity between samples based on their bacterial composition.

A Heatmap showing the Simpson index by participants and surveys was generated. All data were plotted using the ggplot2 package^35^. The relationship between the oropharyngeal microbiome and mucosal immunity (SIgA) was analyzed by Spearman’s correlation.

All data from questionnaires were downloaded from ODK server and transferred into a single Excel spreadsheet, combined with laboratory results. Chi-square or Fisher’s exact tests were used to determine the significance of categorical variables in the questionnaire by Site. Binomial regressions were performed to assess the relationships between bacteria (at genus and phyla level) and dependent variables (Site, Visits and Gender). All analyses were performed using R-4.2.1 software^37^.

## Results

### Participant recruitment and follow up

A total of 39 and 37 participants were enrolled in Abidjan and Korhogo respectively. In Abidjan, one participant dropped out of the study at the second survey (S2) due to lack of consent for sample collection. Additionally, one participant was absent during the last two visits (S5 and S6) from each site.

The cohort gender was skewed towards girls and comprised of 49 (64.5%) girls and 27 (35.5%) boys overall; the gender ratio was more balanced in Korhogo with 19 (51.4%) girls and 18 (48.6%) boys. All the participants were from the required age group of 8–12 years old with most children in the 9- and 10-years old groups at both sites (Table 1).

**Table 1:** Participant demographics and results from the risk factors questionnaire by site.

	Abidjan	%	Korhogo	%
Gender				
Girls	30	76.9	19	51.4
Boys	9	23.1	18	48.6
Total	39		37	
Class level				
CE1 /Grade 2	7	17.9	4	10.8
CE2 /Grade 3	5	12.8	13	35.1
CM1 /Grade 4	12	30.8	13	35.1
CM2 /Grade 5	15	38.5	7	18.9
Total	39		37	
Age (years)				
8	5	12.8	6	16.2
9	10	25.6	18	48.6
10	12	30.8	10	27.0
11	8	20.5	3	8.1
12	4	10.3	0	0
Total	39		37	

	Abidjan (%)	Korhogo (%)	0R (IC)	P-value
Knowledge of meningitis				
High	39.47	37.84	0.93 (0.33–2.61)	0.0103
Low	36.84	10.81	0.21 (0.05–0.79)	
Medium	23.68	51.35	3.34 (1.15–10.39)	
Application of good hygiene measure				
Bad	21.05	2.7	0.1 (0.002–0.87)	0.042
Good	44.74	62.16	2 (0.73–5.66)	
Medium	34.21	35.14	1.04 (0.36-3)	
Nutritional status				
Mild malnutrition	15.79	8.1	0.475 (0.070–2.45)	0.29
Moderate malnutrition	2.63	2.7	1.02 (0.012–82.77)	
Severe malnutrition	0	2.7	Inf (0.036- inf)	
Normal	63.16	81.08	2.46 (0.78–8.46)	
Obesity	5.26	0	0 (0-5.45)	
Excess weight	13.16	5.4	0.38 (0.034–2.53)	
Contact with animals				
Yes	34.21	37.84	1.16 (0.41–3.35)	0.812
No	65.79	62.16		

### Significant geographical differences amongst factors of interest within the cohort

Analysis of responses to the risk factor questionnaire showed some significant differences between the cohorts at both sites (Table 1). Respondents in Abidjan had a higher knowledge of meningitis with the majority classified in the category high knowledge (39.5%) whereas the majority in Korhogo were classified in the category medium knowledge (51.3%): an overall significant difference by χ^2^ test (*p* = 0.0103). No significant difference was found when comparing socioeconomic status of respondents.

Similarly, the application of good hygiene measures (hand washing with soap) was reported to be higher in Korhogo (62.2%) compared to Abidjan (44.7%, *p* = 0.0422). A significant difference in bad hand hygiene practices was observed, with 21.05% in Abidjan compared to 2.7% in Korhogo. No significant differences in the nutritional status of the participants or their contact with animals in relation to the geographical situation, gender, or socioeconomic situation was found.

### Bacterial carriage and mucosal immunity

#### Neisseria carriage

A total of 227 and 220 swabs were collected in Abidjan and Korhogo respectively during the six surveys (S1-S6) but only one *N. meningitidis* carrier (0.45%) was detected by culture at S3 in Korhogo. The same participant was also positive for *N. meningitidis* carriage during S1 by PCR. Culture also identified four episodes of *N. lactamica* carriage and 3 episodes of putative *Neisseria* isolate carriage in Korhogo. One participant was shown to carry *N. lactamica* at 3 different visits.

Overall, 8 episodes of *Neisseria* carriage (3.64%) were identified in Korhogo whereas no *Neisseria* carriage was identified in Abidjan using culture during the whole study (Table 2). No *N. meningitidis* was detected in Abidjan by PCR. This result represents a significant difference in *Neisseria* carriage between both sites (*p* = 0.006).

**Table 2:** Results of *Neisseria* culture and PCR.

		Visits	S1	S2	S3	S4	S5	S6	Total
Abidjan		Participants	39	38	38	38	37	37	227
		*Neisseria meningitidis* (culture)	0	0	0	0	0	0	0
		*Neisseria meningitidis* (RT-PCR/ *ctrA*)	0	0	0	0	0	0	0
		*Neisseria lactamica*	0	0	0	0	0	0	0
		*Neisseria spp.*	0	0	0	0	0	0	0
Korhogo		participants	37	37	37	37	36	36	220
		*Neisseria meningitidis* (culture)	0	0	1	0	0	0	1
		*Neisseria meningitidis* (RT-PCR/ ctrA)	1	0	0	0	0	0	1
		*Neisseria lactamica*	1	0	1	1	0	1	4
		*Neisseria spp.*	0	0	3	0	0	0	3

*S* survey.

When looking at the *Neisseria* carriage by survey, there was a significant difference between proportion of carriers by sites only at survey 3 (January) with a 0% in Abidjan and 13.51% in Korhogo (p value = 0.0253).

### Mucosal immunity variation by survey

**Fig. 2:**
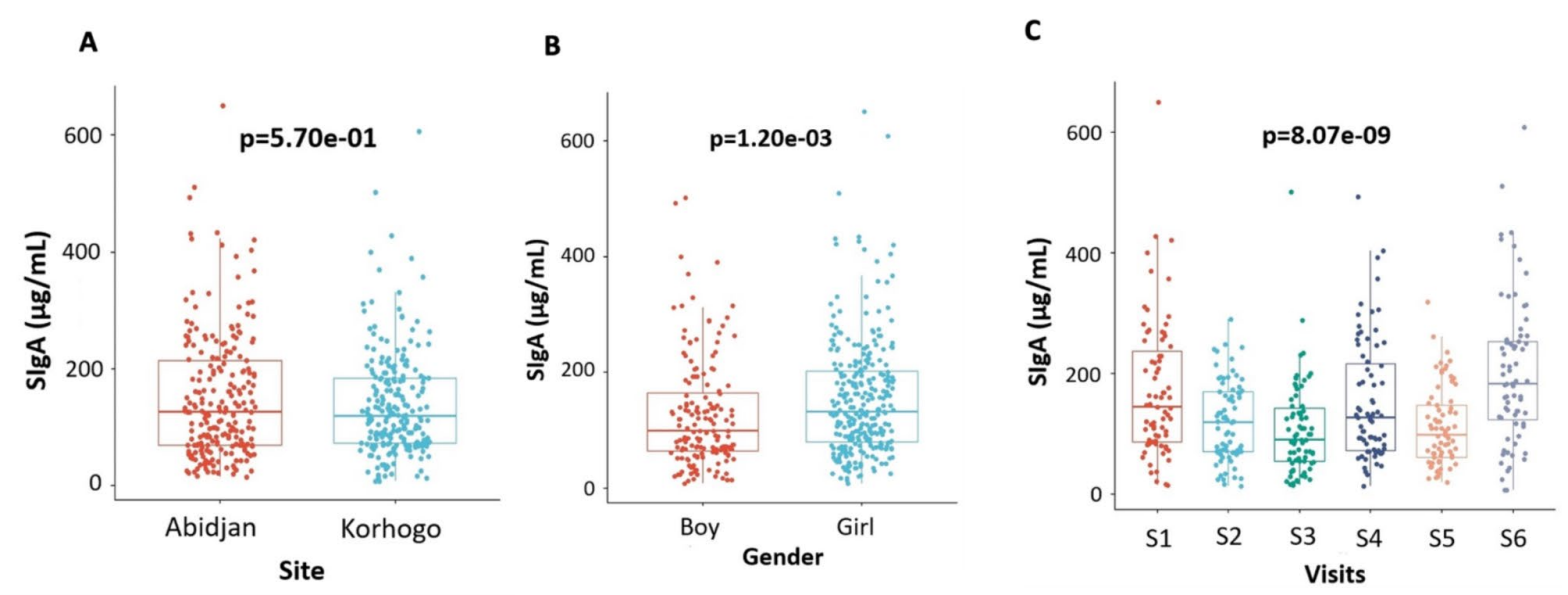
Concentrations of SIgA in children by site (**A**), gender (**B**), and by visit (**C**) during the cohort. The different significance values were determined by Wilcoxon and the Kruskal Wallis test. For the Visits, S is for Survey (S1 = Survey 1).

All children in the cohort had a SIgA concentration higher than minimal threshold of testing at each visit with an overall mean concentration of 144.68 µg/ml. There was no significant difference in mean SIgA concentration by site; however, significant differences were found by gender (Boys = 129.4 µg/ml, Girls = 153.3 µg/ml; *p* = 0.0120) and visit (S1 = 171.82 µg/ml, S2 = 119.11 µg/ml, S3 = 113.10 µg/ml, S4 = 152.58 µg/ml, S5 = 111.37 µg/ml, S6 = 201.46 µg/ml; p < 0.001) (Fig. 2).

### Microbial diversity of the oropharyngeal samples

A total of 227 and 221 DNA samples extracted from swabs collected in Abidjan and Korhogo respectively, were sent for sequencing. Forward and Reverse sequence files were received for all samples sent for Abidjan, however, only 207 pairs of sequences were received for Korhogo. Following QC, and removal of negative controls, 226 and 201 sequences from Abidjan and Korhogo respectively were included in downstream analysis. The positive control was successfully identified, providing confidence in the data integrity.

We targeted the V3-V4 region of the 16 S rRNA gene, and the FASTQ files generated by the Illumina MiSeq platform have an average read length of 287 bases. Regarding read quality, we observed that 66.74% of the sequences had a quality score of 30 or higher, while 29.86% of the sequences had a quality score of 20 or higher. A total of 36,556,031 raw reads were obtained during sequencing. After the filtering and truncation process, 26,384,762 reads were retained. The application of DADA2 for denoising further refined the data to 24,372,298 reads. Following the merging step, 20,465,845 reads remained, which were subsequently refined by removing chimeric sequences, resulting in 18,953,527 high-quality reads. Prior to contamination assessment, a total of 6510 ASVs were identified. After employing the decontam package to identify and eliminate potentially contaminated ASVs, we retained 6499 ASVs. Following the pruning step, the final count of ASVs was reduced to 4240.

**Fig. 3:**
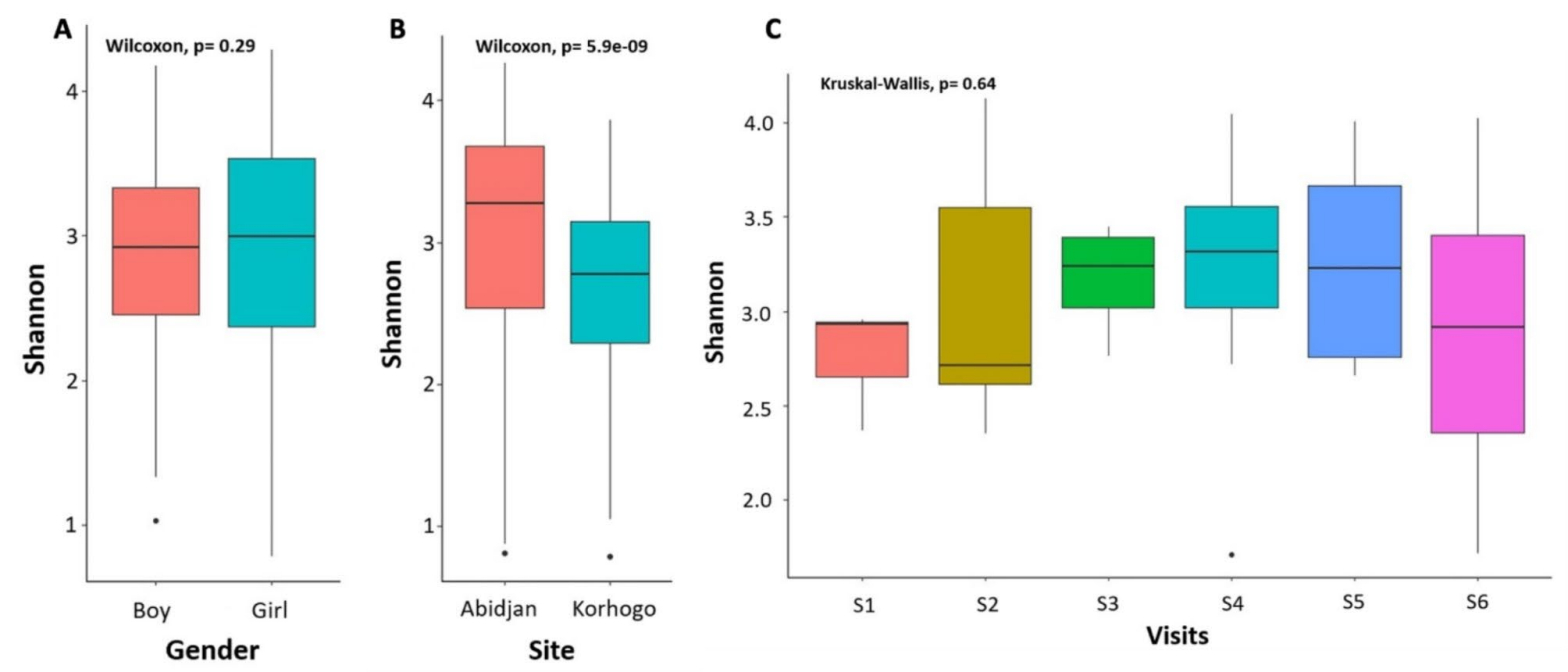
Measure of the Shannon alpha diversity by gender (**A**), Site (**B**) and Visits (**C**). The Wilcoxon Rank Sum test was used for statistical analysis for the pairwise comparison and the Kruskal-Wallis test when more than one variable was being compared. P value ≤ 0.05 was considered significant. For the Visits, S is for Survey (S1 = Survey 1).

The analyses identified 23 different phyla, 312 genera and 199 species. Alpha diversity was significantly different between sites (*p* < 0.001) with a Shannon diversity index higher in Abidjan (mean = 3.3, SD = 0.65) than in Korhogo (mean = 2.7, SD = 0.6). No significant difference was observed by gender or by survey (Fig. 3). No difference in alpha diversity was found when comparing samples based on presence of visible URTI, hygiene level, socio-economic and nutrition status of children or their contacts with animals. A heat map based on the Shannon index revealed this difference in microbial diversity by time and by site for each child (Supplemental Fig. 1).

**Fig. 4:**
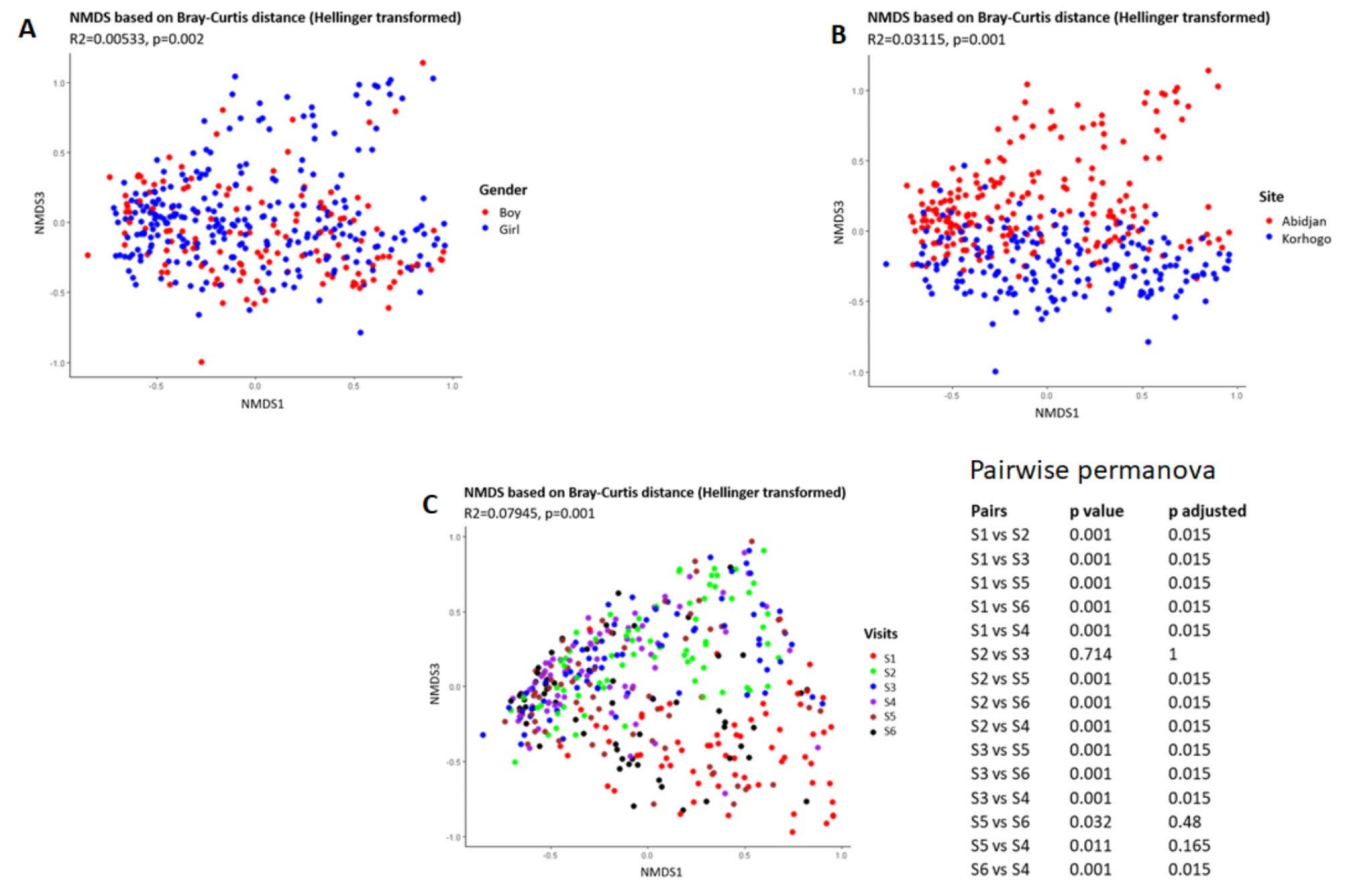
NMDS of the beta diversity of samples based on gender (**A**), Site (**B**) and Visits (**C**). The analysis was based on the Bray Curtis distance. Pairwise Permanova analysis was used to identify significant changes among different visits. P value ≤ 0.05 was considered. significant. For the Visits, S is for Survey (S1 = Survey 1).

Beta diversity, as measured by non-metric multidimensional scaling (nMDS) based on Bray-Curtis distance, confirmed a significant difference in microbial composition between samples from the two sites (*p* = 0.001, PERMANOVA). A significant difference was also observed with respect to gender (*p* = 0.002). Additionally, a significant difference was observed based on visits (*p* = 0.001). A pairwise PERMANOVA analysis revealed that microbial composition varied significantly between all visits except between visits 2 and 3, visits 4 and 5, and visits 5 and 6 (Fig. 4).

The PERMDISP analysis assessed intra-group variability in bacterial composition between Abidjan and Korhogo, revealing that Abidjan had a mean intra-group distance of 0.5635 with a standard deviation (SD) of 0.0744, indicating greater diversity among samples. In contrast, Korhogo had a slightly higher mean distance of 0.5788 but with a lower SD of 0.0685, suggesting more uniform microbial communities (supplemental Table 4). For gender-based analysis, the boy group had a mean intra-group distance of 0.5821 with an SD of 0.0707, while the girl group had a mean distance of 0.5766 and an SD of 0.0694, indicating slightly higher diversity among the boy group samples but with comparable levels of variability between the genders (supplemental Table 5). Among six assessed groups (S1 to S6), Group S2 exhibited the highest average diversity (mean distance = 0.5919) and the lowest variability (SD = 0.0618), whereas group S1 had the lowest average diversity (mean distance = 0.4911) and the highest variability (SD = 0.0909). The other groups displayed intermediate levels of diversity and variability. These findings underscore the differences in microbial community structure and consistency across locations and groups (supplemental Table 6).

**Fig. 5:**
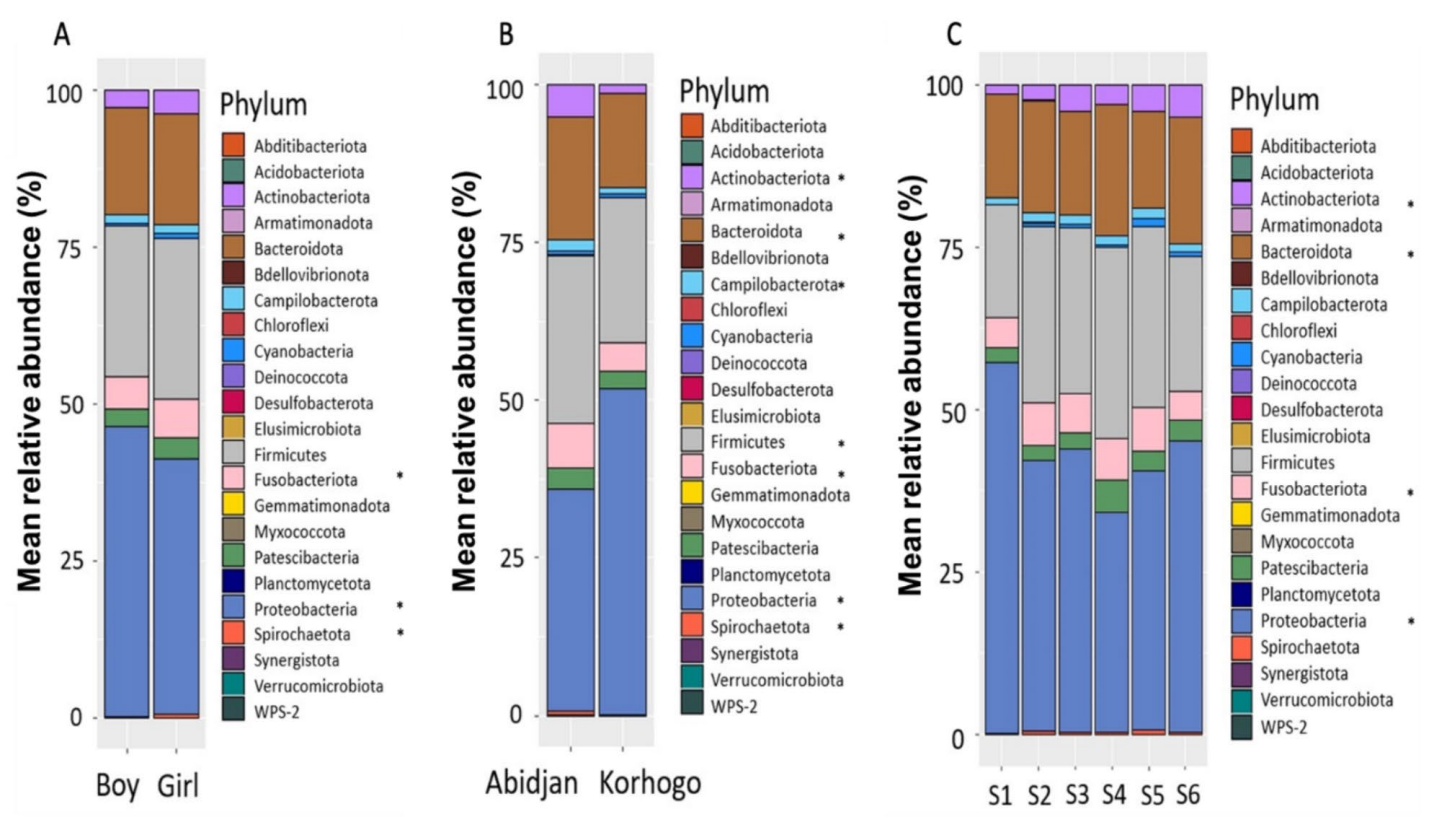
Bacterial diversity and mean relative abundance (%) of microorganisms classified at Phylum level. Distribution by Gender (**A**), Site (**B**) and Visit (**C**). *Corresponds to statistical differences that were considered significant with P value ≤ 0.05 when using a binomial regression model. For the Visits, S is for Survey (S1 = Survey 1).

When considering microorganism distribution at phyla level we found that the most common were Proteobacteria, Firmicutes, Bacteroidota, Fusobacteriota, Actinobacteriota and Patescibacteria. Significant differences (*p* < 0.05) were reported by gender, with Proteobacteria more abundant in boys when compared to girls and, by contrast, Fusobacteriota and Spirochaetota more so in girls. Significant geographical differences were also observed, with Actinobacteriota, Bacteroidota, Campylobacterota, Firmicutes, Fusobacteriota, Spirochaetota more abundant in samples from Abidjan compared to Korhogo, whereas Proteobacteria were more abundant in Korhogo. When comparing phylum variation by visit, 4 showed significant changes over time (p < 0.05): Actinobacteriota, Bacteroidota, Fusobacteriota and Proteobacteria. (Fig. 5, supplemental Table 2).

**Fig. 6:**
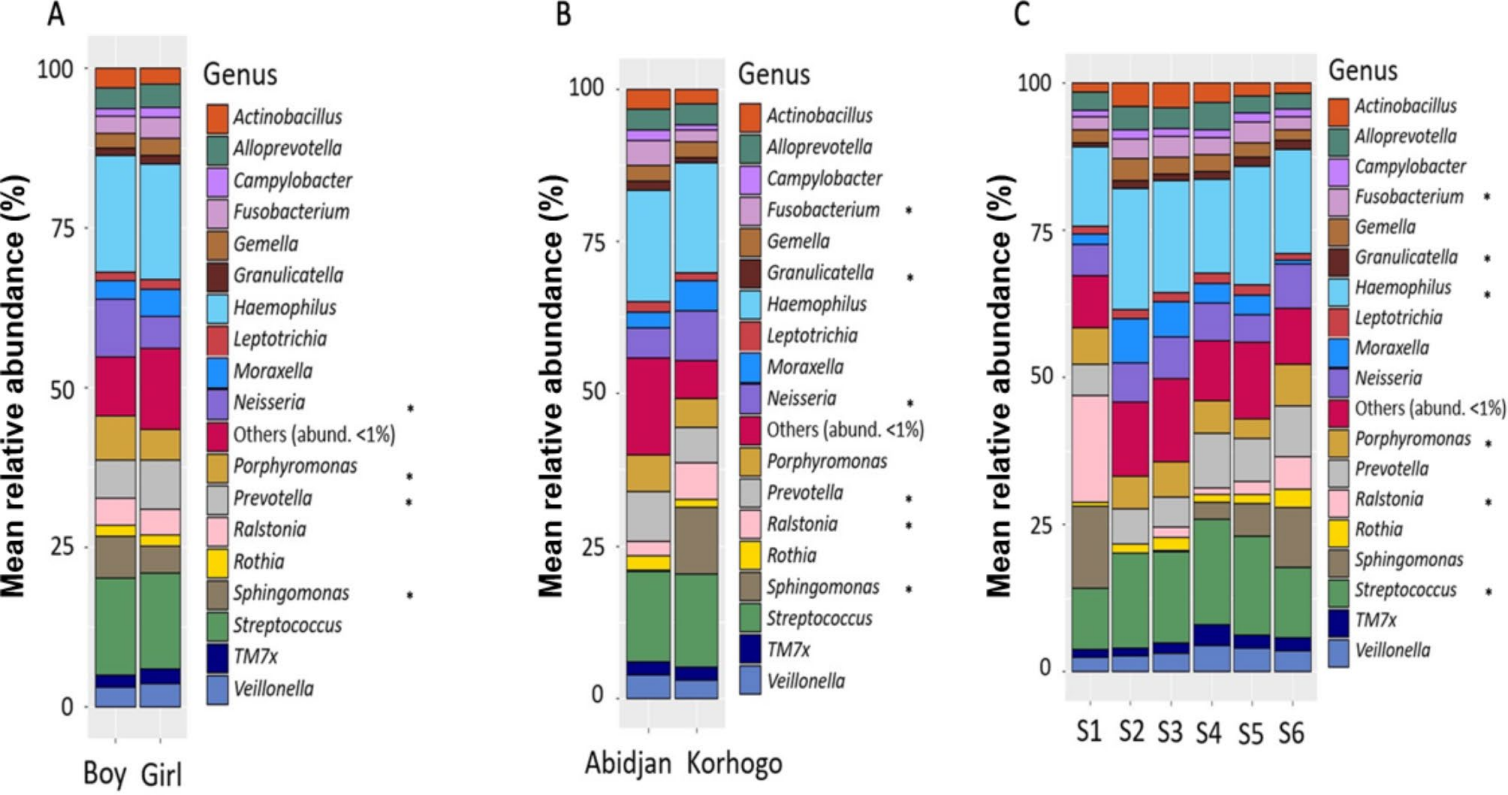
Bacterial diversity and mean relative abundance (%) of microorganisms classified at Genus level. Distribution by Gender (**A**), Site (**B**) and Visit (**C**). The Genus Others correspond to all microorganisms whose relative abundance is lower than 1%. TM7x is also known as genus *Nanosynbacter*. *Corresponds to statistical differences that were considered significant with P value ≤ 0.05 when using a binomial regression model. For the Visits, S is for Survey (S1 = Survey 1).

At the genus level, 18 were detected with a relative abundance above 1%. Some genera showed significant differences when comparing gender; *Neisseria*, *Porphyrromonas*, *Prevotella* and *Sphingonomas* were significantly more abundant in boys when compared to girls (p **<** 0.05). When comparing geographical differences, *Fusobacterium*, *Granulicatella*, *Prevotella* were significantly more abundant in Abidjan compared to Korhogo whereas *Neisseria*, *Ralstonia*, *Sphingomonas* were more abundant in Korhogo (*p* < 0.05). Finally, when looking at temporal differences, 6 genera showed significant changes over time (p < 0.05): *Fusobacterium*, *Granulicatella*, *Haemophilus*, *Porphyromonas*, *Ralstonia* and *Streptococcus* (Fig. 6, supplemental Table 3).

Identification to species level was not possible for all ASV and could be reported for less than 30% of ASV. Two *Neisseria* species could be characterised; *Neisseria flavescens* and *Neisseria mucosa* (supplemental Fig. 2).

### Microbiome and mucosal immunity (SIgA)

**Fig. 7:**
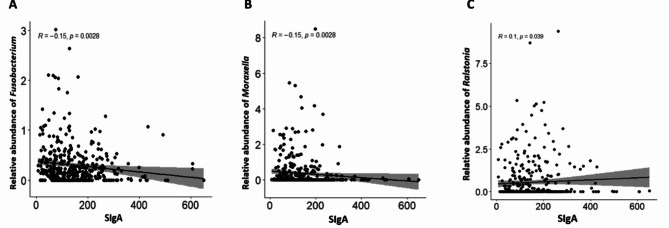
Correlation between relative abundance of genera and mucosal immunity (SIgA). Correlations were found for three genera: *Fusobacterium* (**A**), *Moraxella* (**B**) and *Ralstonia* (**C**). These correlations were established using the Spearman correlation test.

The analyses established a correlation between secretory immunoglobulin A (SIgA) and microbial abundance in the oropharynx. Among the identified genera, three were significantly correlated with SIgA: negative correlations were obtained with the genera *Fusobacterium* (*R*=-0.15; *p* = 0.0028), *Moraxella* (*R*=-0.15; *p* = 0.0028) and a positive correlation with the genus *Ralstonia* (*R* = 0.1; *p* = 0.0390) (Fig. 7).

### Microbiome and climate factors

**Fig. 8:**
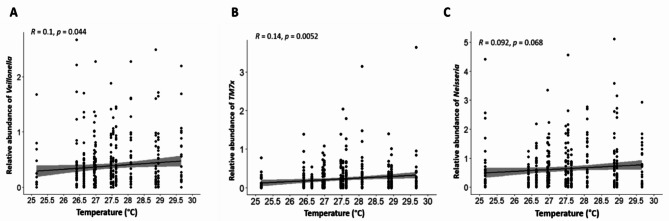
Spearman correlation between Temperature and microbiome diversity. This is depicted in relation to the specific relative abundance of genera *Veillonela* (**A**), *TM7x* (**B**) and *Neisseria* (**C**). TM7x is also known as genus *Nanosynbacter*.

**Fig. 9:**
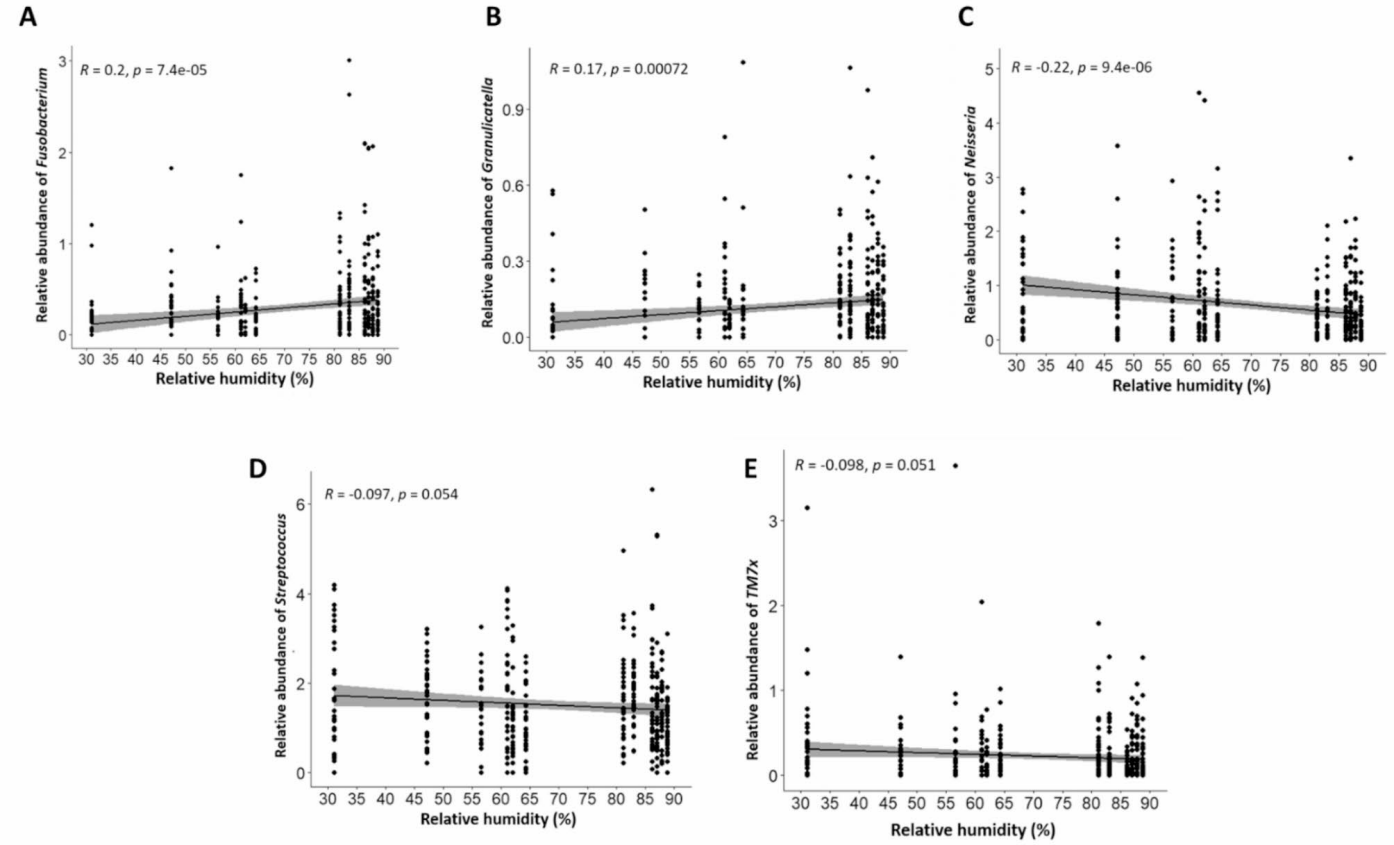
Spearman correlation between relative humidity and microbiome diversity. This is depicted in relation to the specific relative abundance of genera *Fusobacterium* (**A**), *Granulicatella* (**B**) *Neisseria* (**C**) *Streptococcus* (**D**) and *TM7x* (**E**). TM7x is also known as genus *Nanosynbacter*.

**Fig. 10:**
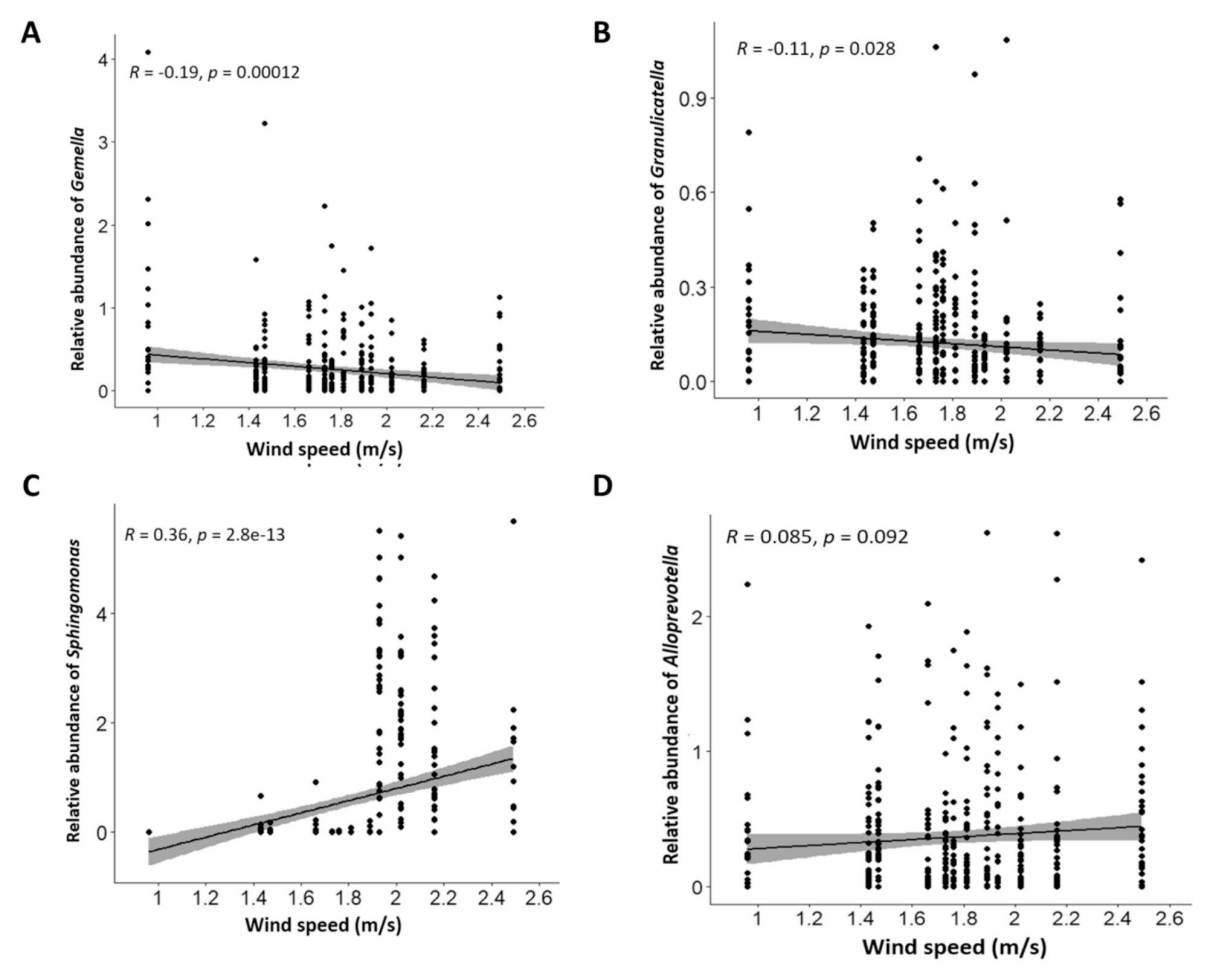
Correlation between Wind speed and microbiome diversity. This is depicted in relation to the specific relative abundance of genera *Gemella* (**A**), *Granulicatella* (**B**) *Sphingonomas* (**C**) and *Alloprevotella* (**D**).

**Fig. 11:**
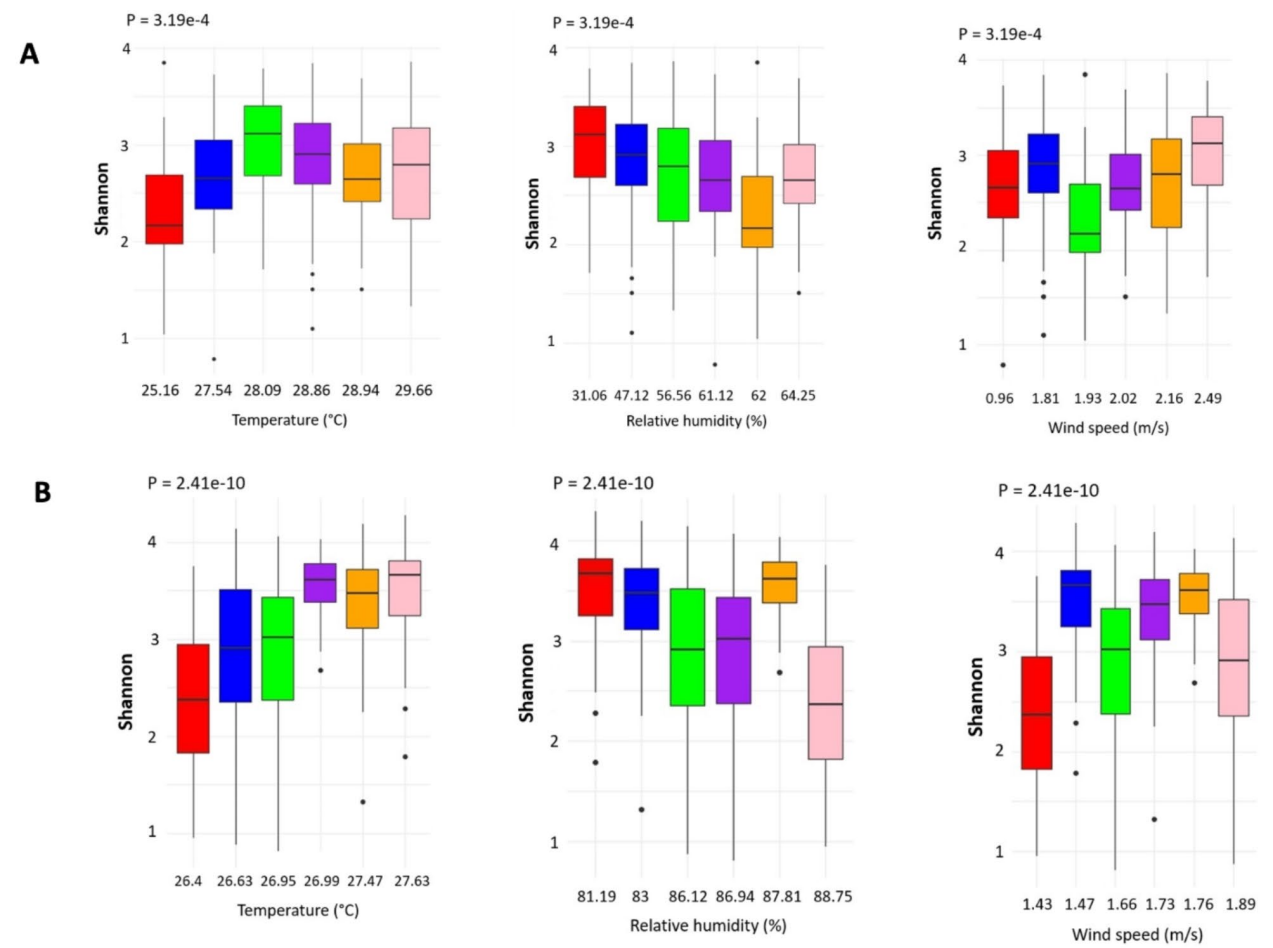
Correlation between meteorological data and microbiome diversity. This is depicted for Temperature, Relative humidity, and Wind speed at each study site, Korhogo (**A**) and Abidjan (**B**). The different significance values were determined by Kruskal Wallis test.

The relationship between the microbiome diversity and climatic factors was investigated for three factors: temperature, humidity, and wind speed. The overall analysis identified significant changes in Shannon diversity index for all three factors using the Kruskal-Wallis rank sum test (Figs. 8A, 9A and 10A). In addition to the overall analysis, these significant differences of the Shannon diversity index associated with changes in weather were also observed at each study site (Fig. 11).

When looking at individual trends in genera, the increase in temperature was significantly associated with higher relative abundance of *Veillonella* and *TM7x* (p < 0.05), a similar trend was also observed for *Neisseria*, but it did not reach significance (Fig. 8B). The increase in relative humidity was also significantly associated with an increased relative abundance of *Fusobacterium* and *Granulicatella* whereas relative abundance of *Neisseria* was significantly reduced (p < 0.001). A reducing trend could also be observed for *Streptococcus* and *TM7x* but did not reach significance (Fig. 9B). Finally, the increase in wind speed associated with a significant decrease in relative abundance of *Gemella* and *Granulicatella* and a significant increase in relative abundance of *Sphingomonas* (p < 0.05). An increasing trend was also observed for *Alloprevotella* but did not reach significance (Fig. 10B).

## Discussion

*Neisseria meningitidis* carriage is a prerequisite for invasive meningococcal disease thus understanding the factors that affect it is important to design preventive measures. *Neisseria meningitis* carriage has been shown to be very dynamic in the African meningitis belt with yearly variation between and within countries^38^ but factors underlying these changes are yet to be completely understood.

Côte d’Ivoire, with the north of the country within the meningitis belt associated with sharp climatic changes and overall lower level of humidity whereas the south of the country is in a more stable and humid climate over the year, presented an ideal setting for comparing factors that could influence *Neisseria meningitidis* in two different meningitis risk contexts. The differences observed in knowledge of meningitis, which was higher in Abidjan than in Korhogo was somewhat unexpected as the latter is situated in the African meningitis belt and therefore its population was expected to be more aware of the disease. However it is important to note that the last big epidemic occurred in 2012^20^ and therefore this could explain why respondents were not more aware than their counterpart in Abidjan. Differences in use of hygiene measures (defined as regular washing of hands with soap) was also identified at a higher rate of application in Korhogo. This may be due to lifestyle differences. Despite being a large city, Korhogo remains less urbanised than Abidjan. Additionally, because this study took place during the Covid-19 pandemic, these results could have been affected by the multiple campaigns for handwashing.

No significant differences in socioeconomic status, nutritional status, frequency of contact with animals among the participants and crowding could be identified when comparing sites or gender. This finding suggests that those factors did not bias the downstream microbiological analysis.

The carriage rate of the meningococcus was very low in the study and therefore made it difficult to assess the impact of factors of interests (geographical, gender and temporal) on that particular bacterium. However, when looking at the *Neisseria* genus overall, a significant geographical difference was found. A geographical difference in *Neisseria* carriage has also been demonstrated outside the African meningitis belt, in South Africa; with a higher incidence of meningococcal disease in provinces with higher carrier rates^39^.

The microbiota analyses based on 16 S rRNA sequencing indicated a larger bacterial diversity (Shannon) in samples coming from Abidjan, the region least at risk of meningococcal epidemic. Higher alpha diversity is often associated with a healthier microbiome with lower relative abundance of pathogenic bacteria^40^. Analysis of the beta diversity which represents the differences between two communities also confirmed this significant geographical difference between both sites. Contrary to the alpha diversity analysis, the nMDS analysis of the beta diversity showed a significant difference when comparing different survey time points of the study suggesting a different microbial structure between these survey time points. This could be explained by a natural temporal dynamic of the oropharyngeal microbiome at certain times points in children. This dynamic could be due to many factors, including maturation during childhood, changes in the diet, medical treatments, or climatic factors^38,39^.

Microbiome analysis allowed the identification of 23 phyla, 312 genera, and 199 species, although species-level identification with V3-V4 region sequencing should be considered with caution, as the resolution of 16 S rRNA sequencing may not always allow precise species delineation. A few studies have worked on the oropharyngeal microbiome and have reported that the healthy microbiome is composed in majority of six phyla: Actinobacteria, Bacteroidetes, Cyanobacteria, Firmicutes, Fusobacteria and Proteobacteria^40^. This study also identified these phyla in addition to *Patescibacteria*. However, *Cyanobacteria* were found to be less abundant than *Patescibacteria*. *Cyanobacteria*, also known as blue-green algae are associated with the lowest ecological zone of water bodies, such as the sea floor (benthic zone), there are two commons routes of human exposure that have been described: through consumption of contaminated water or through recreational activities such as swimming^41^. *Patescibacteria* on the other hand are a recently described new superphyla associated with groundwater^42^. The different lifestyles and the high reliance of populations in Africa on untreated groundwater for water consumption^43^ could explain the higher presence of *Patescibacteria* in this study compared to the commonly described phyla which are mostly derived from studies undertaken in industrialised/higher income countries.

Furthermore, it has been observed that oral microbial diversity is often influenced by individual factors rather than genetic differences between populations, as shown by studies of ethnolinguistic groups in Angola and Zimbabwe, where high levels of pathogenic bacteria have been associated with poor health conditions and compromised immunity in some marginalised communities^44^.

When zooming into the genera level, 18 genera were present at a relative abundance higher than 1% in the study sample and they included families of known potential pathogenic bacteria associated with meningitis such as *Haemophilus*,* Neisseria* and *Streptococcus* amongst others. It is interesting to note that significant differences in relative abundance of *Neisseria* between the two sites was also confirmed with the microbiome analysis, with a larger relative abundance in Korhogo than in Abidjan, similarly to the culture results. A significantly different relative abundance of *Neisseria* by gender was also noted with a larger one in boys compared to girls despite having a study population skewed toward girls. This has been reported before in meningococcal carriage studies conducted within the African meningitis belt^45^. Gender differences have also been associated with bacterial meningitis cases, with male at higher risk of contracting the disease^46,47^. As *Neisseria* is transmitted between individuals through exposure to contaminated body fluid, this gender associated difference may be due to larger social interaction of boys. Another study looking at non-pathogenic *Neisseria* (NPN) found that to the contrary this gender difference was the opposite when considering only NPN^15^. The genera *Streptococcus* and *Haemophilus* varied in relative abundance by survey visits, but not by site or gender, showing temporal differences but not geographical one. An oropharyngeal microbiome study conducted in a college population in the USA identified *Streptococcus*, *Veillonella* and *Rothia* as the most common genera^48^, contrary to this study they used a whole genome metagenomic approach which provided much higher resolution, to the species level. Although *Veillonela* and *Rothia* have been detected in this study they were less abundant than other genera like *Haemophilus*,* Neisseria*,* Porphyromonas* or *Prevotella* which are also known colonisers of the oral cavity. These differences could be due to the geographical and lifestyle differences between the studied populations and to the target age group which was younger (primary school student) in this study compared to the one in the USA (college students).

The 16 S rRNA sequencing analysis presented here identified limited number of species and therefore no statistical analysis was conducted at the species level. It is interesting to note that *Haemophilus influenzae* was the most abundant species detected. In the genus *Neisseria*, only *Neisseria mucosa* and *Neisseria flavescens* could be differentiated; this is in line with previous papers that have established that 16 S rRNA sequencing lack resolution for *Neisseria* species differentiation^49,50^.

High SIgA concentration was observed throughout the cohort in children with significant variation longitudinally as well as by gender. As the different surveys represent different time points during the meningitis season, it is interesting to note that mean SIgA concentrations decrease progressively during the harmattan or meningitis season and increase as the rainy season approaches. Apart from carriage, one study has shown that a decrease of SIgA was an important factor to consider in recurrent infections of the upper airways in children^51^. Because it has been difficult to find an appropriate range to assess the level of SIgA in the literature, the minimal threshold of the test has been used as an indication of low level of SIgA. Another study conducted in Ethiopia showed a significant increase in serogroup specific salivary SIgA levels over time after vaccination with multivalent meningococcal conjugate vaccines^52^. That study examined the immune response after a substantial intervention, vaccination, using an in-house assay and controls and an arbitrary unit, whereas this study measured natural changes in SIgA using a commercial kit with provided controls and unit. Comparisons are therefore difficult to make between those two studies. The role of this salivary secretory immunoglobulin in defence against carriage requires further characterisation.

The study also examined the relationship between microbial relative abundance in the oropharynx and mucosal immunity. Indeed, the relative abundance of genera *Fusobacterium* and *Moraxella* were negatively correlated with SIgA. This suggests that relative abundance of these bacterial genera may promote low mucosal immunity in children. On the contrary, a relative abundance of the genus *Ralstonia* could promote higher mucosal immunity. However, these results must be analysed with caution as they do not tell us anything about directionality of the correlation.

It has been shown that low levels of SIgA in mucous membranes may lead to increased susceptibility to infections^53^. Indeed, the role of SIgA antibodies in the defence against infections is recognised by the fact that most pathogens are first encountered by mucosal membranes^54^. A salivary microbiota study of SIgA-deficient mice revealed a remarkably reduced frequency of *Streptococcus* and increased percentages of *Aggregatibacer*,* Actinobacillus* and *Prevotella* at the genus level compared to wild-type control mice^55^. This suggests that regulation of the respiratory microbiome may play an important role in mucosal immunity.

The correlations between climatic factors (Temperature, Wind speed and Humidity level) and changes in the microbiome were explored in this study and found some significant correlations between changes in the climatic factors and some genera. Of particular interest was the significant correlation between increased humidity level and reduced relative abundance of *Neisseria* species. The positive correlation between temperature and the relative abundance of *Neisseria* did not reach significance. Other genera also had significant correlation with one or more of the climatic factors and further research into the meaning of these correlations would be important to determine the impact of climatic changes on the relative abundance of these different genus and species. Another study that assessed the impact of climatic factors on carriage in the UK focused on a specific bacterium and found a higher odds of *streptococcus pneumoniae* carriage in cooler temperature^56^. A study conducted in Niger on the impact of climate on meningitis disease patterns^57^ found that high temperature was a significant risk for bacterial meningitis and that high temperature and dust inhalation promoted progression from pneumococcal carriage in the nose to invasive disease, in a mouse model. This study concluded that climatic surveillance could be used to predict invasive bacterial diseases epidemics. A study conducted in the Democratic Republic of Congo (DRC) also linked meningococcal carriage and disease with level of humidity and concluded that high absolute humidity seemed to reduce the transmission of meningococci^58^. Indeed, it has been shown that atmospheric conditions such as dry wind and dust can damage the immune barriers of the mucous membrane of the upper respiratory tract and the nasal cavity, which could thus promote meningitis^59,60^. For example, *Neisseria meningitidis* could more easily penetrate damaged mucous membranes through the bloodstream and meninges^61^. Sultan et al. also showed that seasonal winds were a major climatic driver of the meningitis belt, and that a high prevalence of meningitis associated with dry, windy, and dusty conditions^62^.

None of these other studies used a metagenomic approach like this study did and therefore comparison can be difficult. However, it is important to note that the same conclusion in term of links between changes in climatic factors and bacterial relative abundance were found and should therefore be investigated further using larger scale studies at higher genomic resolutions to confirm and help identify mechanistic explanations.

## Conclusion

This study provides an unbiased analysis of the oropharyngeal microbiome in a cohort of school children in Côte d’Ivoire. It identified significant differences between the two sites of the study both in terms of meningitis associated bacterial carriage but also in alpha and beta diversity of the microbiota. The low carriage of *Neisseria meningitidis* did not allow to study the impact of the different factors of interest on the bacterium. Variations in secretory SIgA were observed throughout the cohort with a link with changes in abundance of certain bacterial genera in the oropharyngeal microbiome. Correlations between climatic factors and abundance of specific bacteria were also identified as significant and require further investigation.

## Electronic supplementary material

Below is the link to the electronic supplementary material.

Supplementary Material 1

Supplementary Material 2

Supplementary Material 3

Supplementary Material 4

## Data Availability

The sequence data are available on the NCBI Sequence Read Archive with accession numbers ranging from SRX23222127 to SRX23221695; https://www.ncbi.nlm.nih.gov/sra/PRJNA1064912 . All other data used in this manuscript (database in csv and scripts) are included in the manuscript as supplementary information files. The Scripts are available in Supplemental document 1 and the Database available in Supplemental document 2.

## Abbreviations

ASV: Amplicon sequence variant
CNESVS: Comité national d’éthique des sciences de la vie et de la santé [National Ethics Committee for Life Sciences and Health
CRICK: The Francis Crick Institute
CSRS: Centre Suisse de Recherches Scientifiques en Côte d’Ivoire
DNA: Deoxyribonucleic acid
EDTA: Ethylenediaminetetraacetic acid
GGA: Gamma Glutamyl Aminopeptidase
LANADA: Laboratoire National d’Appui au Développement Agricole
NEB: New England Biolabs
ODK: Open Data Kit
ONPG: O-nitrophenyl-beta-D-galactopyranoside
PCR: Polymerase Chain Reaction
PNSSU: Programme National de la Santé Scolaire et Universitaire [National school and university health program
RNA: Ribonucleic acid
SigA: Secretory Immunoglobulin A
URTI: Upper respiratory tract infection
WACCBIP: West African Centre for Cell Biology of Infectious Pathogens
WGS: Whole-Genome Sequencing
WHO: World Health Organization
