# Supplementary material for: Spatiotemporal dynamics of the oropharyngeal microbiome in a cohort of Ivorian school children
Source: Sci Rep. 2024 Dec 28;14:30895. doi: 10.1038/s41598-024-81829-6 (PMC11681117; doi:10.1038/s41598-024-81829-6)
Supplement: Supplementary file 3 — Supplementary Material 3 [file 41598_2024_81829_MOESM3_ESM.docx]

**Spatiotemporal dynamics of the oropharyngeal microbiome in a cohort of Ivorian school children**

**Supplemental Figures**


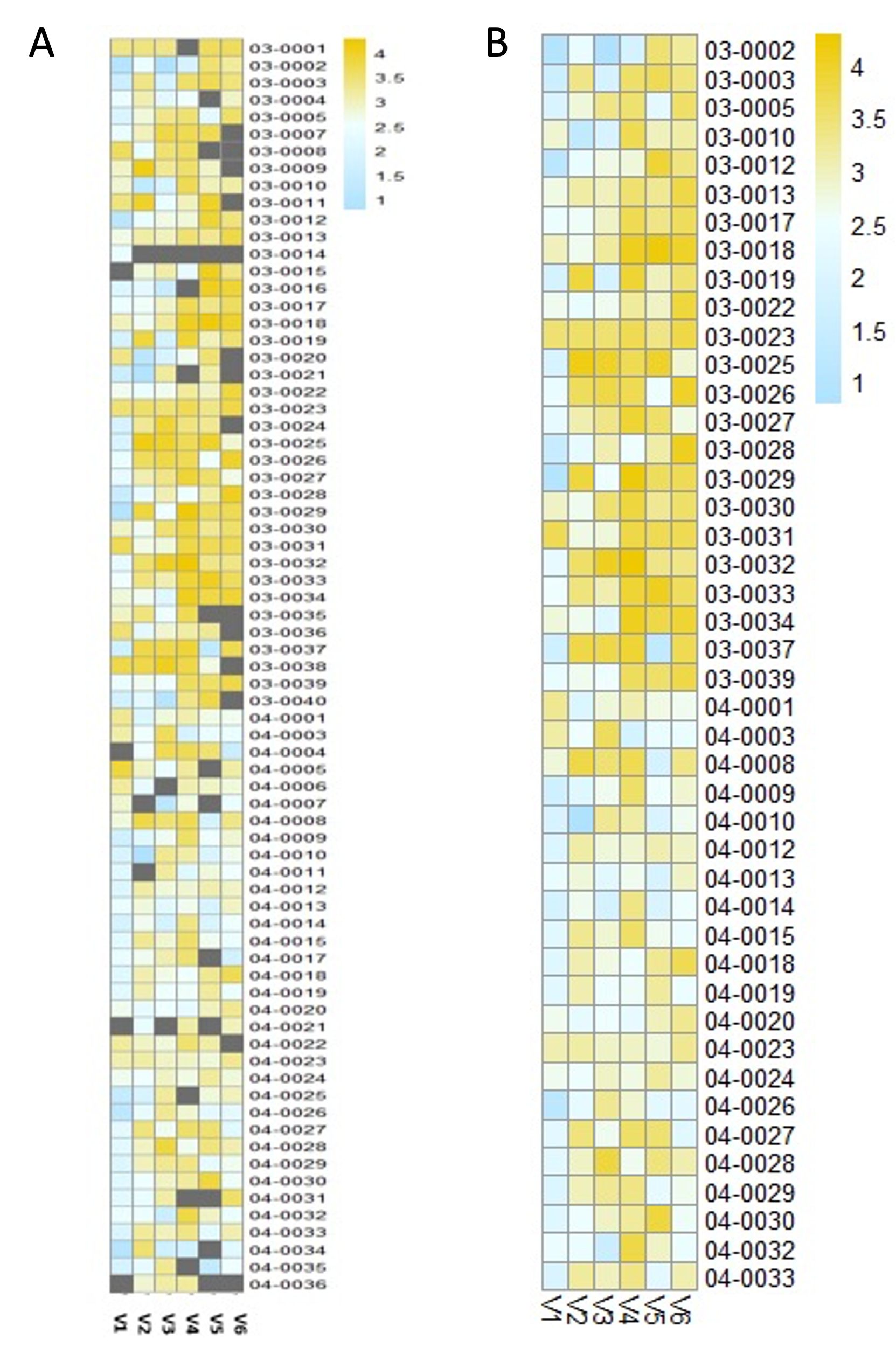


**Supplemental figure 1**: Heat map illustrating the evolution of the shannon alpha- diversity over time (S1-S6) for all participants (A) and for participants for which we had microbiome data for all 6 time points (B). The grey boxes in (A) correspond to the missing data points. IDs beginning with 03 represent participants in Abidjan and those beginning with 04 participants in Korhogo.


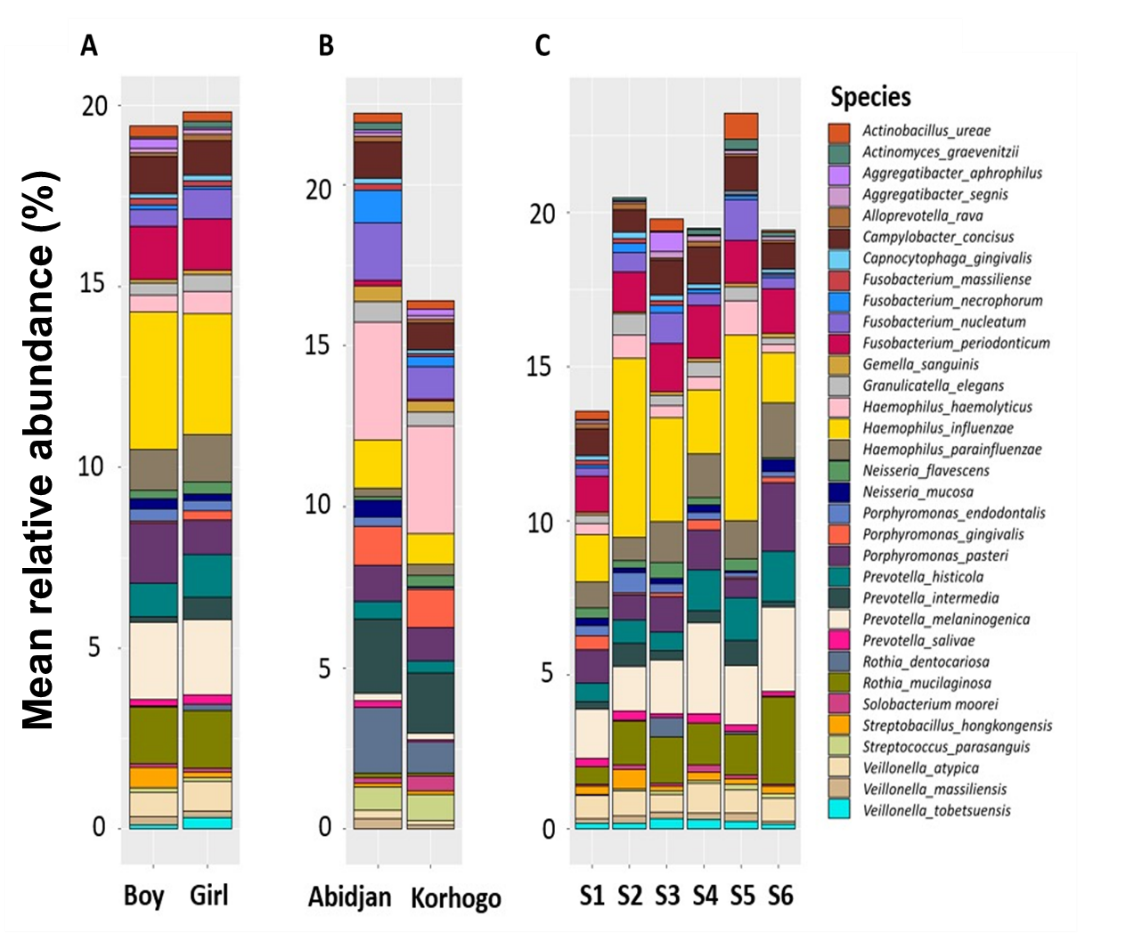


**Supplemental Figure 2**: Bacterial diversity and mean relative abundance (%) of microorganisms classified at species level. Distribution by Gender (A), Site (B) and Visit (C).

**Supplemental Tables**

**Supplemental table 1:** Risk factors questionnaire

| **Question #** | **Identification Question** | **Response** |
| --- | --- | --- |
| **1.** | Study Identifier | 0 3-_ _ _ _ |
| **2.** | Date of Questionnaire Administration [D/M/Y] | _ _ /_ _ /_ _ _ _ |
| **3.** | Participant's Gender | Girl Boy |
| **4.** | Date of Birth [D/M/Y] | _ _ /_ _ /_ _ _ _ |
|  | **Socio-Economic Aspect** |  |
| **5.** | How many people live in your house? | # |
| **6.** | How many bedrooms are there in your house? | # |
| **7.** | How many toilets are there in your house? | # |
| **8.** | Where are the toilets located in your house? | Inside Outside |
| **9.** | Are there smokers in your house? | No Yes |
| **10.** | Where is the kitchen in your house? | Inside Outside |
| **11** | What fuel do you use for cooking? (Multiple answers can be circled) | Gas  Electricity  Kerosene  Charcoal  Wood  Straw  Cow Dung |
| **12.** | What is your mode of transportation to and from school? | Personal Car  Personal Motorcycle  Public Transport (Taxi, gbaka, wôrô wôrô…)  Walking |
| **13.** | What is your father's occupation? | Primary Sector (Agriculture, Livestock)  Secondary Sector (Industries) Tertiary Sector (Service) Unemployed |
| **13.b** | What is your father's education level? | Primary  Secondary  High School  University  No Education |
| **14.** | What is your mother's occupation? | Primary Sector (Agriculture, Livestock)  Secondary Sector (Industries) Tertiary Sector (Service) Unemployed |
| **14.b** | What is your mother's education level? | Secondary  High School  University  No Education |
|  | **Environmental Aspect** |  |
| **15.** | Do you have pets at home? | No Yes |
| **15.a** | If yes, which ones? | Dog  Cat  Chicken  Sheep  Goat  Cow  Horse  Other: ____________ |
| **15.b** | Do your pets enter the house? | No Yes |
| **15.c** | Where are these animals kept? | None  Enclosure  Yard  Other: … |
| **15.d** | Who in the family takes care of these animals? | Children  Father  Mother  Everyone |
| **16.** | Do you consume cow's milk at home | No Yes |
| **16.b** | If yes, is it boiled (pasteurised)? | No Yes |
|  | **Health and Nutrition Aspect** |  |
| **17.** | Are you aware of meningitis? | No Yes |
| **17.b** | If yes, are you afraid of this disease? | No Yes |
| **17.c** | If yes, do you think it is a deadly disease? | No Yes |
|  | If yes, do you think it is a contagious disease? | No Yes |
| **18.** | Have you been vaccinated against meningitis? | No Yes |
| **18.a** | Do you have your vaccination card? | No Yes |
| **18.b** | Can the answer to question 18 be confirmed?  What meningitis vaccine was taken? | No Yes  ____________________________ |
| **19.** | Weight in kg |  |
| **20.** | Height in cm |  |
| **21.** | Measurement of forearm circumference in cm |  |
| **22.** | Which meals do you have every day?  Breakfast  Lunch  Snack  Dinner | No Yes  No Yes  No Yes  No Yes |
| **23.** | Do you share your meals with family in a common bowl? | Always  Often  Rarely  Never |
| **24.** | Do you wash your hands with soap before all your meals? | Always  Often  Rarely  Never |
|  |  |  |
|  |  |  |
|  |  |  |
|  |  |  |

**Supplemental table 2:** Relative abundance of the most common phyla by Site, Survey and Sex


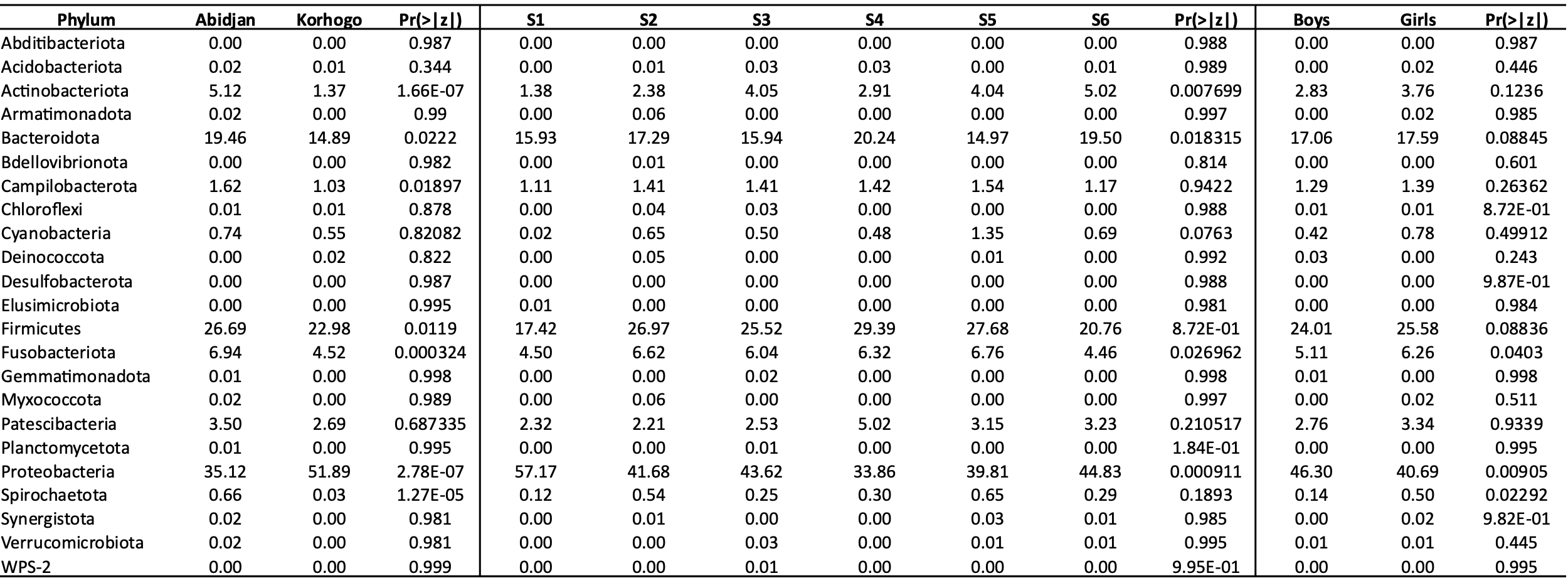


**Supplemental table 3:** Relative abundance of the most common genera by Site, Survey and Sex

| **Genus** | **Abidjan** | **Korhogo** | **Pr(>\|z\|)** | **S1** | **S2** | **S3** | **S4** | **S5** | **S6** | **Pr(>\|z\|)** | **Boys** | **Girls** | **Pr(>\|z\|)** |
| --- | --- | --- | --- | --- | --- | --- | --- | --- | --- | --- | --- | --- | --- |
| *Actinobacillus* | 3.19 | 2.29 | 0.0703 | 1.41 | 3.93 | 4.17 | 3.16 | 2.13 | 1.61 | 0.08041 | 3.09 | 2.62 | 0.272 |
| *Alloprevotella* | 3.57 | 3.42 | 0.3023 | 3.24 | 4 | 3.44 | 4.63 | 2.89 | 2.8 | 0.35626 | 3.22 | 3.66 | 0.9474 |
| *Campylobacter* | 1.61 | 1.03 | 0.0655 | 1.11 | 1.41 | 1.4 | 1.41 | 1.51 | 1.17 | 0.51889 | 1.28 | 1.38 | 0.4189 |
| *Fusobacterium* | 4.02 | 1.81 | 1.10E-5 | 2.01 | 3.47 | 3.62 | 2.96 | 3.52 | 2.28 | 0.01724 | 2.55 | 3.29 | 0.2763 |
| *Gemella* | 2.6 | 2.65 | 0.9944 | 2.37 | 3.69 | 2.74 | 2.69 | 2.51 | 1.74 | 0.42038 | 2.41 | 2.74 | 0.8879 |
| *Granulicatella* | 1.62 | 0.93 | 2.75E-5 | 0.67 | 1.39 | 1.12 | 1.4 | 1.54 | 1.5 | 0.00237 | 1.13 | 1.40 | 0.0785 |
| *Haemophilus* | 18.17 | 17.95 | 0.75617 | 13.45 | 20.56 | 19 | 15.89 | 20.14 | 17.87 | 0.0195 | 18.17 | 18.01 | 0.955779 |
| *Leptotrichia* | 1.72 | 1.3 | 0.98454 | 1.33 | 1.55 | 1.63 | 1.94 | 1.72 | 0.96 | 0.1041 | 1.34 | 1.63 | 0.347371 |
| *Moraxella* | 2.71 | 5.03 | 0.07648 | 1.71 | 7.46 | 5.85 | 3.26 | 3.29 | 0.64 | 0.0706 | 2.92 | 4.22 | 0.352626 |
| *Neisseria* | 4.94 | 8.08 | 8.28E-5 | 5.35 | 6.56 | 7.21 | 6.38 | 4.75 | 7.58 | 0.0754 | 9.02 | 4.89 | 7.41E-5 |
| *Others (abund. <1%)* | 15.84 | 6.27 |  | 8.87 | 12.66 | 14.21 | 10.27 | 12.91 | 9.55 |  | 9.38 | 12.71 |  |
| *Porphyromonas* | 6.16 | 4.8 | 0.123917 | 6.11 | 5.54 | 5.84 | 5.53 | 3.42 | 7.06 | 0.0119 | 6.87 | 4.82 | 0.0156 |
| *Prevotella* | 8.03 | 5.84 | 0.033038 | 5.31 | 5.98 | 5.08 | 9.18 | 7.37 | 8.57 | 0.0931 | 5.96 | 7.64 | 0.0224 |
| *Ralstonia* | 2.48 | 6.03 | 1.17E-4 | 18.32 | 0.05 | 1.82 | 1.1 | 2.13 | 5.61 | 6.22E-11 | 4.21 | 4.00 | 0.0866 |
| *Rothia* | 2.36 | 1.12 | 0.282855 | 0.6 | 1.52 | 2.23 | 1.43 | 1.52 | 3.04 | 0.3059 | 1.71 | 1.85 | 0.691 |
| *Sphingomonas* | 0.1 | 11.11 | 4.42E-6 | 13.99 | 0.05 | 0.18 | 2.83 | 5.6 | 10.16 | 0.1115 | 6.61 | 4.21 | 0.0201 |
| *Streptococcus* | 14.86 | 15.28 | 0.672 | 10.37 | 16.03 | 15.6 | 17.97 | 16.89 | 11.95 | 6.87E-5 | 15.13 | 15.00 | 0.5408 |
| *TM7x* | 2.18 | 2.02 | 0.287 | 1.35 | 1.34 | 1.7 | 3.52 | 2.19 | 2.26 | 0.0697 | 1.88 | 2.23 | 0.7736 |
| *Veillonella* | 3.84 | 3.04 | 0.186 | 2.44 | 2.81 | 3.16 | 4.45 | 3.98 | 3.64 | 0.1548 | 3.09 | 3.70 | 0.1035 |

**Supplemental table 4:** Intra-Group Variation in Sample Distances by Site

| Site | Mean Intra-Group Distance | SD Intra-Group Distance |
| --- | --- | --- |
| Abidjan | 0.5635 | 0.0744 |
| Korhogo | 0.5788 | 0.0685 |

This table summarizes the intra-group variation in sample distances for two groups: Abidjan and Korhogo. The mean intra-group distance provides an indication of the average dissimilarity between samples within each group. For Abidjan, the mean distance is 0.5635, while for Korhogo, it is slightly higher at 0.5788, suggesting that the samples from Korhogo are slightly more diverse in composition.

The standard deviation (SD) of intra-group distance gives a measure of how much variability there is in these distances. A higher SD indicates more variability in the distances between samples within the group. In this case, Abidjan has a slightly higher SD (0.0744) compared to Korhogo (0.0685), suggesting that there is more variability between samples in the Abidjan group than in the Korhogo group. This difference could reflect varying degrees of sample heterogeneity within each group.

**Supplemental table 5**: Intra-Group Variation in Sample Distances by Gender

| Gender | Mean Intra-Group Distance | SD Intra-Group Distance |
| --- | --- | --- |
| Boy | 0.5821 | 0.0707 |
| Girl | 0.5766 | 0.0694 |

This table presents the intra-group variation in sample distances for the Boy and Girl groups. The mean intra-group distance represents the average dissimilarity between samples within each group. The Boy group has a slightly higher mean distance (0.5821) compared to the Girl group (0.5766), indicating a marginally greater diversity among samples in the Boy group.

The standard deviation (SD) of intra-group distance reflects the variability within each group. A higher SD suggests more variability in distances between samples. In this case, both groups show similar variability, with the Boy group having an SD of 0.0707 and the Girl group an SD of 0.0694, indicating that both groups exhibit relatively comparable levels of intra-group diversity.

### Supplemental table 6: Intra-Group Variation of Sample Distances by Visits

| Visits | Mean Intra-Group Distance | SD Intra-Group Distance |
| --- | --- | --- |
| S1 | 0.4911 | 0.0909 |
| S2 | 0.5919 | 0.0618 |
| S3 | 0.5893 | 0.0697 |
| S4 | 0.5404 | 0.0661 |
| S5 | 0.5711 | 0.0809 |
| S6 | 0.5411 | 0.0834 |

This table shows the intra-group variation in sample distances across six groups (S1 to S6). The **mean intra-group distance** reflects the average dissimilarity between samples within each group, while the **standard deviation (SD)** indicates the variability in these distances.

- **S2** exhibited the highest mean distance (0.5919) with the lowest SD (0.0618), suggesting the highest average diversity and relatively consistent intra-group composition.
- **S3** had a similar mean distance (0.5893) but with a higher SD (0.0697), indicating comparable diversity with greater variability.
- **S5** and **S6** showed moderate diversity with SDs of 0.0809 and 0.0834, respectively, indicating a moderate level of variability.
- **S1** had the lowest mean distance (0.4911) with the highest SD (0.0909), reflecting the least diversity but the highest variability among samples.
- **S4** had a mean distance of 0.5404 and an SD of 0.0661, showing moderate diversity and variability.
