# Supplementary material for: Spatiotemporal dynamics of the oropharyngeal microbiome in a cohort of Ivorian school children
Source: Sci Rep. 2024 Dec 28;14:30895. doi: 10.1038/s41598-024-81829-6 (PMC11681117; doi:10.1038/s41598-024-81829-6)
Supplement: Supplementary file 4 — Supplementary Material 4 [file 41598_2024_81829_MOESM4_ESM.docx]

Script for the analyses of the 16S RNA sequences

# Load required libraries

library(dada2)

library(decontam)

library(here)

library(phyloseq)

# Set path to data

path <- "~/DK lab"

# List all Fastq files

fns <- list.files(path)

# Create lists for forward and reverse Fastq files

list_R1_fastq <- list.files(path, pattern = "R1_001.fastq$|R1_001.fastq.gz$", full.names = TRUE)

list_R2_fastq <- list.files(path, pattern = "R2_001.fastq$|R2_001.fastq.gz$", full.names = TRUE)

# Number of files

n_files <- length(list_R1_fastq)

n_files

# Extract sample names

fnFs <- sort(list.files(path, pattern="_R1_001.fastq"))

fnRs <- sort(list.files(path, pattern="_R2_001.fastq"))

sample.names <- sapply(strsplit(fnFs, "_L001"), `[`, 1)

# Specify full path to the fnFs and fnRs

fnFs <- file.path(path, fnFs)

fnRs <- file.path(path, fnRs)

# Plot quality profiles

plotQualityProfile(fnFs[[1]])

plotQualityProfile(fnFs[[2]])

plotQualityProfile(fnRs[[1]])

plotQualityProfile(fnRs[[2]])

# Create directory and filenames for filtered Fastq

filt_path <- file.path(path, "filtered")

if(!file_test("-d", filt_path)) dir.create(filt_path)

filtFs <- file.path(filt_path, paste0(sample.names, "_F_filt.fastq.gz"))

filtRs <- file.path(filt_path, paste0(sample.names, "_R_filt.fastq.gz"))

# Filter

for(i in seq_along(fnFs)) {

fastqPairedFilter(c(fnFs[i], fnRs[i]),

c(filtFs[i], filtRs[i]),

truncLen=c(270,270),

trimLeft = 20,

maxN=0,

maxEE=c(5,10),

truncQ=2,

rm.phix=TRUE,

compress=TRUE,

matchIDs = TRUE,

verbose=TRUE)

}

# Remove non-existing files

exists <- file.exists(filtFs)

filtFs <- filtFs[exists]

exists <- file.exists(filtRs)

filtRs <- filtRs[exists]

# Dereplicate Fastq files

derepFs <- derepFastq(filtFs, verbose=TRUE)

derepRs <- derepFastq(filtRs, verbose=TRUE)

# Name derep-class objects by sample names

names(derepFs) <- sample.names[exists]

names(derepRs) <- sample.names[exists]

# Learn error rates

errF <- learnErrors(filtFs, multith=TRUE)

errR <- learnErrors(filtRs, multith=TRUE)

plotErrors(errF)

plotErrors(errR)

# Denoise reads

dadaFs <- dada(derepFs, err=errF, multith=TRUE)

dadaRs <- dada(derepRs, err=errR, multith=TRUE)

# Merge paired reads

mergers <- mergePairs(dadaFs, filtFs, dadaRs, filtRs, verbose=TRUE)

seqtabAll1 <- makeSequenceTable(mergers)

table(nchar(getSequences(seqtabAll1)))

# Remove chimeras

seqtabNoC1 <- removeBimeraDenovo(seqtabAll1)

# Create tracking table

getN <- function(x) sum(getUniques(x))

track1 <- cbind(sapply(dadaFs, getN), sapply(dadaRs, getN), sapply(mergers, getN), rowSums(seqtabNoC1))

colnames(track1) <- c("denoisedF ", "denoisedR", "merged", "nonchim")

rownames(track1) <- sample.names

head(track1)

write.csv(track1, "recap1.txt")

# Taxonomic assignment

fastaRef1 <- "silva_nr99_v138_train_set.fa.gz"

taxTab1 <- assignTaxonomy(seqtabNoC1, refFasta = fastaRef1, multith=TRUE)

taxa1 <- addSpecies(taxTab1, "silva_species_assignment_v138.fa.gz")

taxa.print1 <- taxa1 # Removing sequence rownames for display only

rownames(taxa.print1) <- NULL

head(taxa.print1)

# Define ranks of interest

ranks <- c("domain", "phylum", "class", "order", "family", "genus", "species")

colnames(taxa1) <- ranks

rownames(taxa1) <- getSequences(seqtabNoC1)

write.table(taxa1, "assigntaxonomy1.tsv", sep = "\t")

# Create phyloseq object before decontamination

map <- import_qiime_sample_data("meta.tsv")

ps <- phyloseq(otu_table(seqtabNoC1, taxa_are_rows = FALSE), sample_data(map), tax_table(taxa1))

# Add tree

random_tree <- rtree(ntaxa(ps), rooted=TRUE, tip.label=taxa_names(ps))

ps <- merge_phyloseq(ps, random_tree)

# Define negative controls

negative_controls <- c("03-S1-Blank-1_S15", "03-S1-Blank-2_S42", "03-S1-Control_S41",

"03-S2-Control_S81", "03-S4-Control_S160", "03-S5-Control_S200",

"03-S6-Control_S237", "Blank-PCR_S95")

neg <- sample_names(ps) %in% negative_controls

# Define positive controls

positive_controls <- c("Pos-Ctl-Ecoli_S96", "Pos-Ctl-Ecoli-PLT2_S192")

# Detect contaminants with a threshold of 0.05

contamDF <- isContaminant(ps, neg = neg, threshold = 0.05)

# Filter contaminant ASVs

ps <- prune_taxa(!contamDF$contaminant, ps)

# Remove negative and positive controls

positive_controls <- c("Pos-Ctl-Ecoli_S96", "Pos-Ctl-Ecoli-PLT2_S192")

p <- subset_samples(ps, !(sample_names(ps) %in% c(negative_controls, positive_controls)))

p

# Prune taxa with less than 10 counts across all samples

pp <- prune_taxa(taxa_sums(p) > 10, p)

# Remove samples with 0 total counts

pp1 <- prune_samples(sample_sums(pp) > 0, pp)

# Keep only samples with at least 1000 reads

pp2 <- prune_samples(sample_sums(pp1) >= 1000, pp1)

# Remove taxa that have 0 counts after the previous filtering steps

pp2 <- prune_taxa(taxa_sums(pp2) > 0, pp2)

pp2 # Final phyloseq object after all filtering steps
